# Lipid-based nanosystems: the next generation of cancer immune therapy

**DOI:** 10.1186/s13045-024-01574-1

**Published:** 2024-07-19

**Authors:** Ziyun Cheng, Seth-Frerich Fobian, Elena Gurrieri, Mohamadreza Amin, Vito Giuseppe D’Agostino, Mojtaba Falahati, Sara Zalba, Reno Debets, María J. Garrido, Mesha Saeed, Ann L. B. Seynhaeve, Hayri E. Balcioglu, Timo L. M. ten Hagen

**Affiliations:** 1 Precision Medicine in Oncology (PrMiO), Department of Pathology, Erasmus MC Cancer Institute, Erasmus Medical Center, Rotterdam, The Netherlands; 2 Nanomedicine Innovation Center Erasmus (NICE), Erasmus Medical Center, Rotterdam, The Netherlands; 3 Laboratory of Biotechnology and Nanomedicine, Department of Cellular, Computational and Integrative Biology (CIBIO), University of Trento, Trento, Italy; 4 Department of Pharmaceutical Sciences, School of Pharmacy and Nutrition, University of Navarra, Pamplona, Spain; 5 Instituto de Investigación Sanitaria de Navarra (IdiSNA), Navarra Institute for Health Research, Pamplona, Spain; 6 Laboratory of Tumor Immunology, Department of Medical Oncology, Erasmus MC Cancer Institute, Erasmus Medical Center, Rotterdam, The Netherlands

**Keywords:** Liposomes, Lipid nanoparticles, Immunotherapy, Nanoimmunotherapy, Nanomedicine, Adoptive cellular therapy, Immune checkpoint inhibition, Tumor microenvironment, Nucleic acid delivery, Biomimetic nanosystems

## Abstract

Immunotherapy has become an important part of the oncotherapy arsenal. Its applicability in various cancer types is impressive, as well as its use of endogenous mechanisms to achieve desired ends. However, off-target or on-target-off-tumor toxicity, limited activity, lack of control in combination treatments and, especially for solid tumors, low local accumulation, have collectively limited clinical use thereof. These limitations are partially alleviated by delivery systems. Lipid-based nanoparticles (NPs) have emerged as revolutionary carriers due to favorable physicochemical characteristics, with specific applications and strengths particularly useful in immunotherapeutic agent delivery. The aim of this review is to highlight the challenges faced by immunotherapy and how lipid-based NPs have been, and may be further utilized to address such challenges. We discuss recent fundamental and clinical applications of NPs in a range of areas and provide a detailed discussion of the main obstacles in immune checkpoint inhibition therapies, adoptive cellular therapies, and cytokine therapies. We highlight how lipid-based nanosystems could address these through either delivery, direct modulation of the immune system, or targeting of the immunosuppressive tumor microenvironment. We explore advanced and emerging liposomal and lipid nanoparticle (LNP) systems for nucleic acid delivery, intrinsic and extrinsic stimulus-responsive formulations, and biomimetic lipid-based nanosystems in immunotherapy. Finally, we discuss the key challenges relating to the clinical use of lipid-based NP immunotherapies, suggesting future research directions for the near term to realize the potential of these innovative lipid-based nanosystems, as they become the crucial steppingstone towards the necessary enhancement of the efficacy of immunotherapy.

**Graphical abstract:**

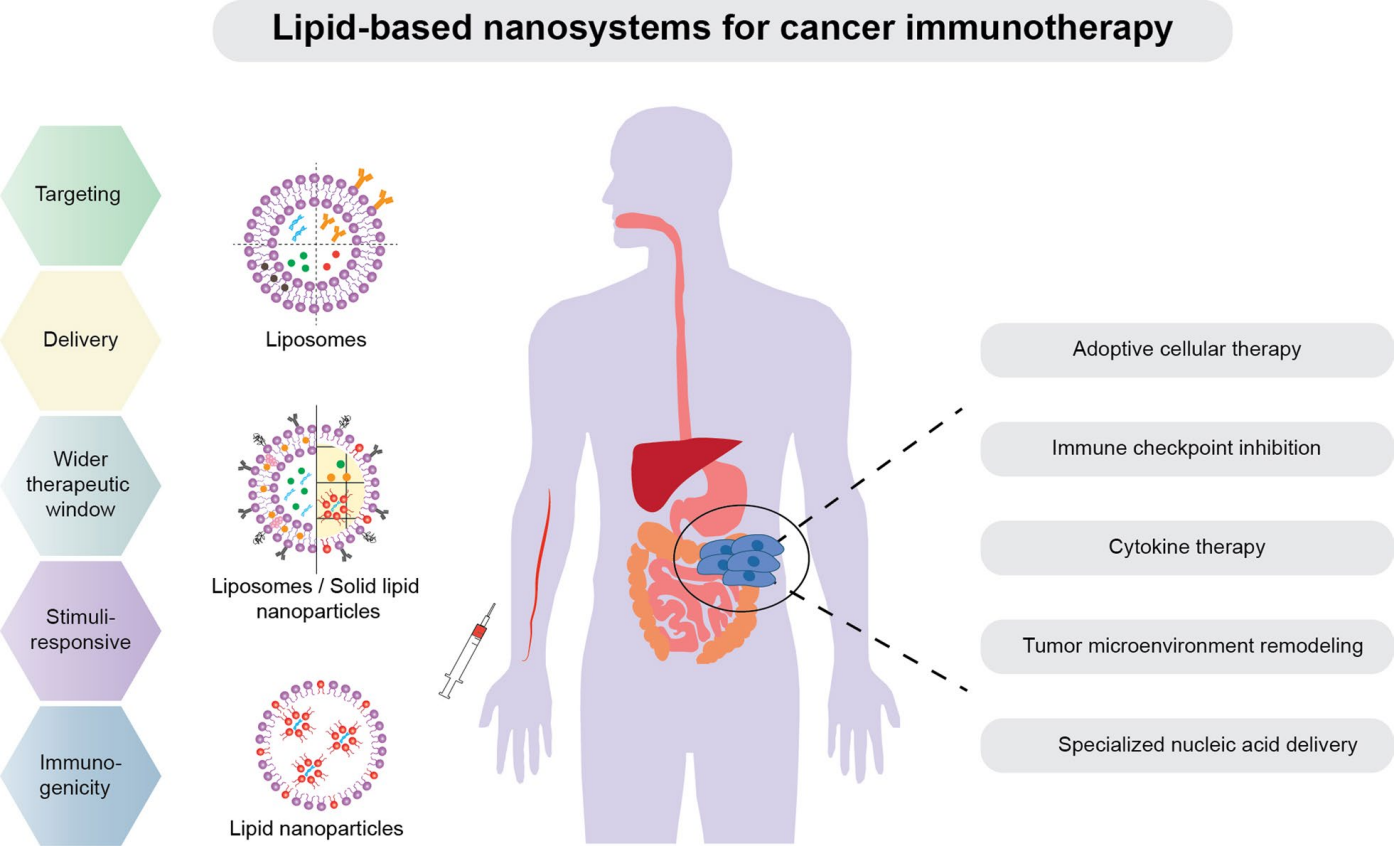

## Background

Cancer immunotherapy aims to reprogram local and systemic immune responses to achieve tumor elimination [1]. However, immunotherapy is frequently accompanied by severe adverse events and does not always result in a durable clinical response, introducing major therapeutic obstacles [2]. In an effort to overcome these obstacles and improve treatment safety and efficacy, the utilization of nanoparticles (NPs) as drug delivery systems is one of the most promising modalities, given their multifunctionality and tailorability.

**Fig. 1:**
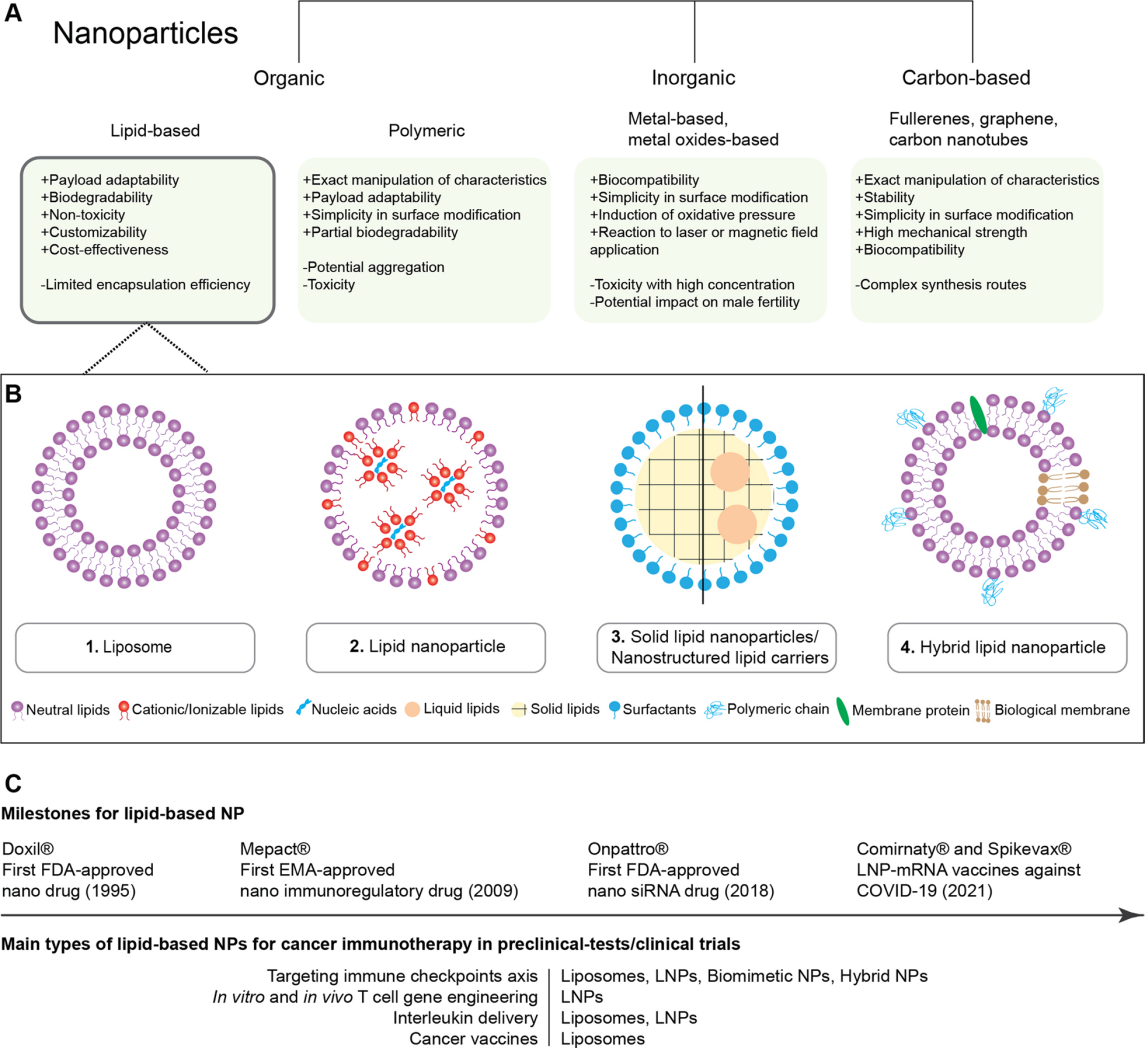
Classification of nanoparticles and lipid-based nanosystems. **A** Nanoparticles (NP) consist of organic NPs (two representative types are lipid-based NPs, polymeric NPs), inorganic NPs and carbon-based NPs. **B** Lipid-based nanosystems include: 1. Liposomes contain a phospholipid bilayer and aqueous core. 2. Lipid nanoparticles (LNPs) are typically used for nucleic acid delivery, consisting of cationic/ionizable lipids**.** 3. Solid lipid nanoparticles can be stabilized with a fully crystallized lipid core (left), while nanostructured lipid carriers are made with the solid lipids partially substituted with liquid lipids for enhanced nanoparticle storage stability and drug loading capacity (right). 4. Hybrid lipid nanoparticles integrate other functional materials such as polymers or biological membrane components to achieve multifunctionality. **C** Lipid-based nanoparticles (NPs) have emerged as one of the most extensively studied drug delivery systems, with many striking developments in recent decades. Various formulations such as liposomes, LNPs, biomimetic NPs, and hybrid NPs have shown impressive positive outcomes in the field of cancer immunotherapy

The term NPs defines objects with all dimensions in the nanoscale (1–100 nm) [3], and they can be categorized into organic [4], carbon-based, and inorganic NPs [5–7], based on their composition (Fig. 1A) [8, 9]. Among these, lipid-based nanoparticles to transport immunotherapeutic agents, are most widely used and explored [10]. The typical lipid-based drug delivery nanosystems consist of liposomes, lipid nanoparticles (LNPs), solid lipid nanoparticles (SLNs), nanostructured lipid carriers (NLCs, nanoemulsions), and hybrid lipid nanoparticles (Fig. 1B) [11, 12]. Liposomes are spherical vesicles composed of one or multiple bilayers of amphiphilic phospholipid molecules that surround an internal aqueous core. LNPs are liposome-like structures, which are widely used for nucleic acid delivery, with multilayer cores due to interactions between negatively charged nucleic acids and cationic lipids. SLNs are colloidal carriers composed of a solid lipid core surrounded by a layer of amphiphilic surface-active molecules. For SLNs, better storage stability and drug loading capacities can be achieved by partially substituting these with liquid lipids (NLCs). Additionally, a large range of hybrid lipid-based NPs are being explored in order to address the limitations of single-component particles, by integrating other materials such as polymers or cell membranes [13, 14].

Clinically, liposomes and LNPs have proven track records [15], with Doxil^®^ (liposomal doxorubicin) being the first Food and Drug Administration (FDA) approved nano-drug (1995) and Onpattro^®^ (small interfering ribonucleic acid (siRNA) encapsulated in LNPs against amyloidosis), being the first FDA approved siRNA therapeutic (2018) [16, 17]. Several lipid-based NP drugs designed specifically for immunological applications have also received approvals from FDA and European Medicines Agency (EMEA). Examples include Mepact^®^, a liposome formulated with the immunomodulatory peptide-lipid mifamurtide, and a nucleotide-binding oligomerization domain containing 2 (NOD2)-agonist, the latter activating monocytes and macrophages against tumor cells [18] as well as Comirnaty^®^ and Spikevax^®^, the LNP-mRNA vaccines against COVID-19 [19]. These pioneering examples facilitated research in a wider focal area, spanning infectious diseases [20, 21], autoimmune diseases [22], and importantly, cancer (Fig. 1C) [23]. The combination of nanosystems with immunotherapy generates synergy, not only pharmacologically and therapeutically, but also for the clinical advancement of both fields.

Building upon the past achievements of lipid-based nanosystems, which have been the most successful of all nanomedicines thus far, key successes of NP-immunotherapeutics are expounded, and emerging applications within this field are discussed. To maintain sufficient depth and conciseness, the scope of this review is limited, as far as possible, to a discussion of research on lipid-based NPs with immunotherapeutic applications. While lipid-based therapeutic cancer vaccines have also become promising modalities for immunotherapeutic cancer elimination, a detailed discussion of vaccination strategies, which is a large and diverse field, is outside of the defined scope of this article. For more information and in-depth analyses of therapeutic nanovaccine systems, interested readers are referred to recent topical reviews [24–29]. Several related review articles have been published in a variety of reputable peer-reviewed cancer-focused [30], immunology-focused [10], and nanotherapy-focused journals [31]. Upon inspection of these papers, it was noted that they have either taken a broader stance on nanotherapy as a whole [32], have chosen to focus on several classes of nanoparticles/nanostructures [33, 34], or specifically focus on a single disease or treatment modality [35–38]. The absence and need of an in-depth review article in the burgeoning field of nano-immunotherapy necessitates this unique and highly relevant review. We believe this review will pique interest in understanding and advancing the applications of lipid-based NPs in combination with immunotherapies.

## Advantages offered by lipid-based nanosystems

**Fig. 2:**
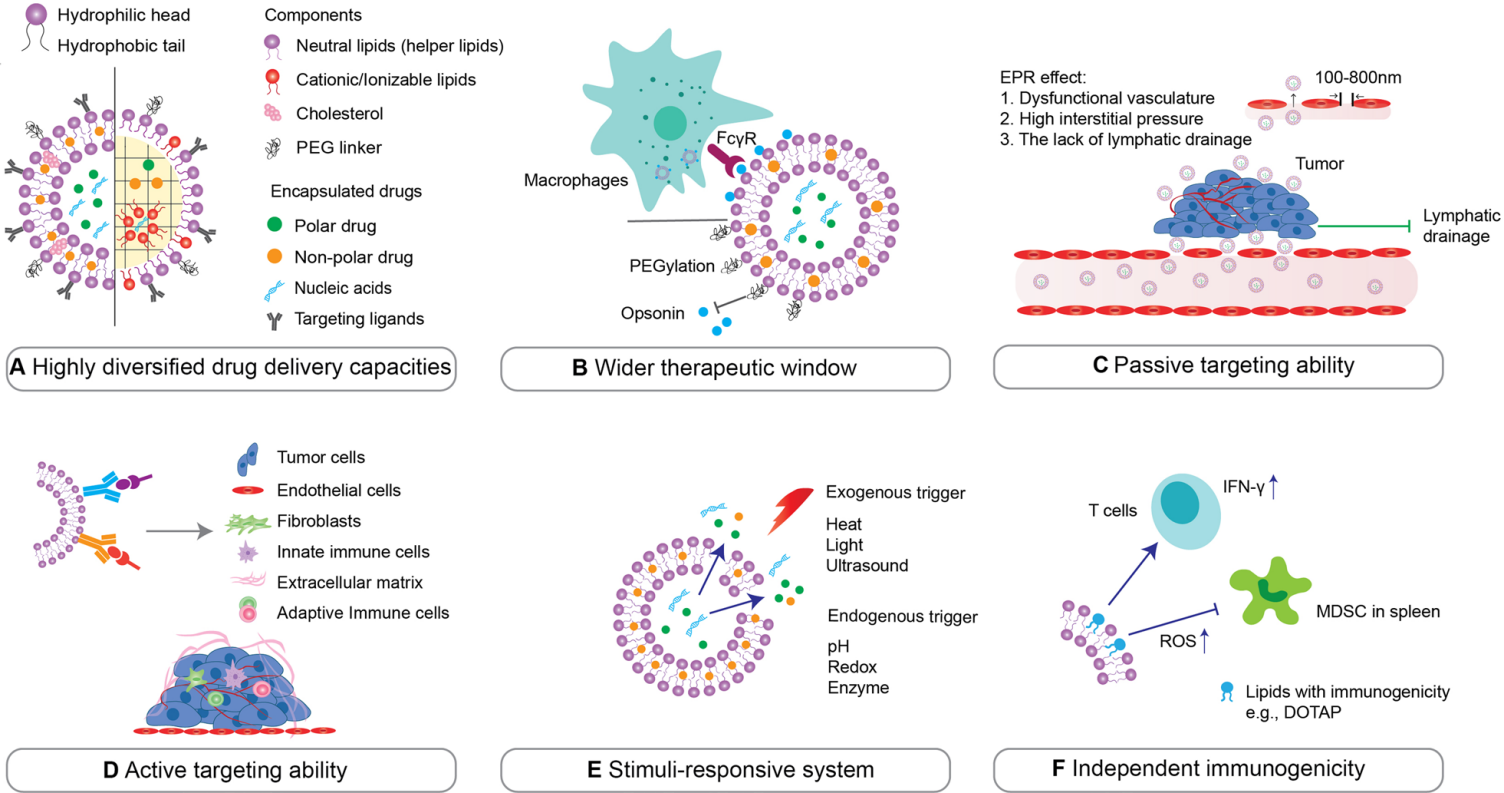
Advantages of lipid-based nanosystems. **A** Lipid-based nanoparticles (NPs; left: liposome. Right: lipid nanoparticle) have wide-ranging drug-loading capacities for the delivery of therapeutic agents including polar and non-polar small-molecule drugs, and macromolecules such as proteins and nucleic acids. The formulations thereof can also be altered for desired biological outcomes; e.g., cholesterol is used for adjusting membrane fluidity, elasticity and permeability. **B** Drug encapsulation by NPs leads to a wider therapeutic window through the protection of cargo against degradation, clearance reduction and systemic exposure reduction. In particular, surface-anchoring of NPs with polyethylene glycol (PEG) can avoid recognition of NPs via opsonin tagging; thus, reducing clearance by the mononuclear phagocytic system (MPS). **C** Passive targeting to cancers occurs via the enhanced permeability and retention (EPR) effect, based on the characteristics of the tumor microenvironment (TME). **D** Active targeting ligands can be attached externally, giving NPs the ability to target and accumulate at specific tissues and cell types. **E** Exogenous triggers, including hyperthermia, magnetism, light, and ultrasound, or endogenous triggers, including pH, redox, and enzymes, can all be used to influence the release of cargos or behavior of lipid-based NPs in the bloodstream, or upon reaching the target site. **F** Particular lipids can themselves have immune-stimulatory effects, thus extending lipid-based NPs’ functions beyond only delivery. Abbreviations: *DOTAP* 1,2-dioleoyl-3-trimethylammonium-propane, *ROS* reactive oxygen species, *IFN* interferon, *MDSC* myeloid-derived suppressor cell

In the last three decades, immunotherapeutics utilizing lipid-based nanotechnology have been instrumental in improving anti-tumor drug administration [10, 39]. The following series of advantages, together, account for their enhanced clinical efficacy (Fig. 2).

### Delivery capabilities

Firstly, lipid-based NPs enable simultaneous delivery of various types of agents, including both hydrophobic and hydrophilic small molecule drugs, antibodies, and nucleic acids (Fig. 2A). This is achieved by leveraging the amphiphilic and responsive properties of lipid molecules; i.e., loading hydrophobic molecules within the lipid bilayer or the lipid core, and hydrophilic molecules within the aqueous core of a liposome, as well as surface adsorption or covalent conjugation of a variety of molecules on lipid-based NPs [40, 41], of which many examples are discussed within this article. In this way, drugs with poor aqueous solubility or low permeability, which would show low bioavailability become administrable, and these challenges can be overcome [42]. Additionally, the diversified drug delivery capacities of NPs facilitate synergistic activity of two or more agents with incompatible pharmacokinetics (PK), e.g., spatiotemporal co-delivery of both antigens and adjuvants, or immunogenic cell death (ICD)-inducing drugs with immunotherapeutic antibodies [43].

In addition to diversified delivery capacities, lipid-based NPs can enhance the PK of payloads through a reduction of clearance rate and reducing the distribution volume while protecting the payload against degradation (Fig. 2B) [17, 44]. Encapsulation of cytotoxic agents within the lipid bilayer or aqueous core of liposomes can significantly reduce systemic exposure and thereby lower non-specific dose-limiting toxicity, widening the therapeutic window [44]. While NPs generally tend to remain contained within the vasculature system better than free compounds, their effectiveness can be limited by opsonization, clearance, and destruction via the mononuclear phagocytic system (MPS) [45]. This crucial challenge can be addressed by optimizing the colloidal, morphological, and surface characteristics: for instance, polyethylene glycol (PEG)-modified liposomes can largely avoid clearance by MPS, thereby improving concentration of the drug in blood compared to non-PEGylated counterpart [46]. Moreover, improvement of PK also comes from the size of NPs: small enough to increase circulation time and reduce the likelihood of phagocytosis, large enough to avoid glomerular filtration and renal clearance (size cut-off: 200–500 nm (spleen), 50–100 nm (liver), 6–8 nm (renal)) [47].

### Passive accumulation, active targeting, and controlled release

Another well-known advantage of NPs is their ability to accumulate in the tumor microenvironment (TME) and other inflamed areas due to the enhanced permeability and retention (EPR) effect (Fig. 2C) [48–52]. This phenomenon is predominantly influenced by altered regional blood flow to the tumor, permeability of the tumor vasculature, structural barriers imposed by perivascular tumors, stromal cells, and extracellular matrix (ECM), as well as intratumoral pressure, adding tumor accumulation as a key site of accumulation next to, primarily, the liver [53]. This effect can be specific to certain tumor types in some cases, and in other cases, is insufficient for a targeted effect. Thus, further specific tissue and cellular targeting of NPs can be achieved by their decoration with tissue-specific ligands (Fig. 2D). This is referred to as active targeting, whereby a specific interaction is utilized between the tumor cell (or tumor-associated feature) and the NP for therapy or accumulation. Not only does this increase targeted accumulation within areas of interest, but it also, importantly, decreases the interactions of these NPs with other tissues. In our experience, this is a powerful way of increasing the therapeutic effects at lower doses, thereby lowering the toxicity of the delivered payload and ensuring a targeted effect. This has primarily been achieved by antibody-fragment or peptide-mediated targeting of unique features on either tumor cells or tumor vasculature [54–69].

NPs can also be engineered to release their payload in a controlled manner (e.g., via ultrasound, light, and hyperthermia), allowing spatiotemporal manipulation for controlled and targeted drug release (Fig. 2E) [70–73]. However, similar “control” may also stem from endogenous triggers. Certain recently developed ionizable/cationic lipids, used in cationic liposomes and LNPs, respond to pH changes in more acidic subcellular compartments, which is essential for membrane fusion, enabling endosomal escape during subcellular trafficking [74]. Even specific features of the TME such as the redox status and certain enzymes produced therein can be exploited to help to achieve site-specific NP delivery and content release [75].

### Immunogenic potential

Lastly, lipid-based NPs, such as liposomes, are generally acknowledged for their biocompatibility and tendencies to evoke minimal immune responses when compared to alternative drug delivery systems. However, their specific immunological impacts may vary based on factors like their composition, size, surface properties, and interactions with the immune system. When there is a need to modulate the immunogenicity, this can indeed be accomplished through the utilization of various components, like adjuvant lipids. For instance, Nikpoor et al. [76] showed that 1,2-dioleoyl-3-trimethylammonium-propane (DOTAP) and 1,2-dioleoyl-sn-glycero-3-phosphoethanolamine (DOPE)-containing liposomes can induce cluster of differentiation 8^+^ (CD8^+^) effector T cells (cytotoxic T lymphocytes, CTLs) to secret interferon (IFN)-γ, promoting the immune response (Fig. 2F). Taheri et al. [77] found that DOTAP-containing liposomes can reduce myeloid-derived suppressor cell (MDSC) populations in the spleen, potentially removing a measure of immunosuppression. This elevates the NPs from delivery mechanisms to immune-modulators to achieve synergistic effects. While this feature has been utilized in lipid-based nanotherapies, immunogenicity can understandably also become a drawback, posing risks for other side effects.

As mentioned above, PEGylation has been a popular technique for shielding NPs in circulation for decades. However, it is not without its drawbacks. Within five days of administration, B cells begin to produce a measurable amount of anti-PEG IgM antibodies (known as the induction phase) [78–80]. This is followed by the effector phase, in the event of another administration 5–21 days after the first dose, whereby the IgM binds PEG, initiates complement binding, and facilitates its removal by the MPS, termed accelerated blood clearance (ABC) [81]. One approach to address this is by achieving saturation at the second administration, as well as pre-administration, or “priming” the circulation with non-functional PEG [82, 83]. Other methods to prolong the circulation of PEGylated NPs and avoid the ABC effect have also been suggested. One such avenue of research to overcome this effect is the development of cell membrane-coated NPs [84], or NP-extracellular vesicle hybrids [85]. This allows coated NPs to gain some of the long circulating (> 120 days) capabilities of, for instance, red blood cells (RBCs), through the display of antiphagocytic signal molecules, one of which being CD47, and the existence of an exterior glycocalyx on these cells [84]. RBC membrane-coated NPs have shown up to 50-day circulation in vivo [86, 87]. A wide range of other cell membrane functionalizations for diverse applications have been explored recently [88, 89], and have been discussed within the context of immune therapy in later sections of this paper.

## Immunotherapy with lipid-based nanosystems

**Fig. 3:**
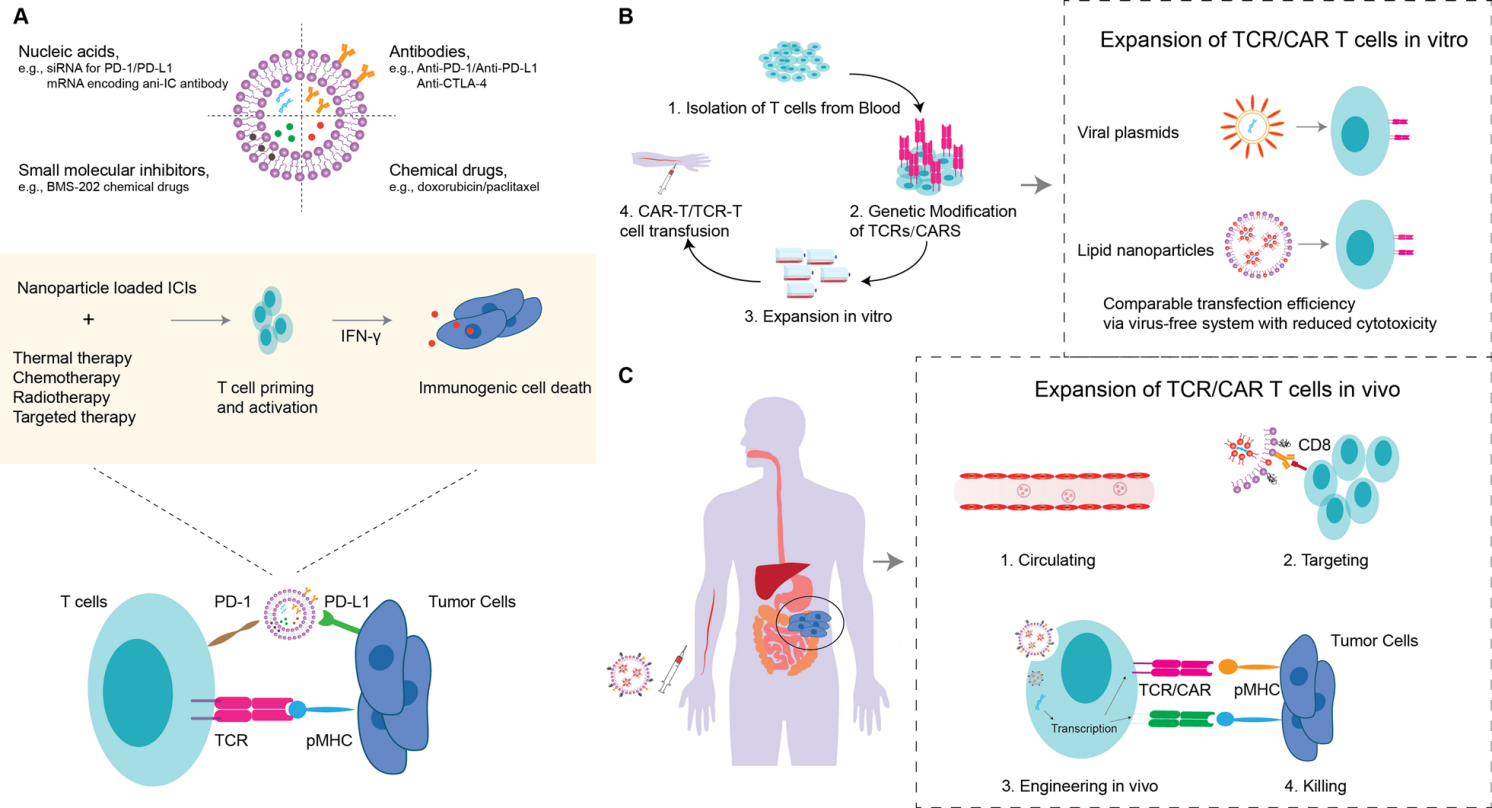
Immunotherapy with lipid-based nanosystems. **A** Nanoparticles (NPs) can deliver diverse types of molecules to achieve immune checkpoint therapy, including antibodies, nucleic acids and small molecular inhibitors. NPs delivering single or multiple checkpoint inhibitor therapies can be combined with thermal therapy, chemotherapy, radiotherapy, or combinations thereof, to enhance immunogenic cancer cell death. **B** Genetically modified T cell receptor (TCR) or chimeric antigen receptor (CAR) T cells can be generated using lipid-based NPs, e.g., lipid nanoparticles, achieving comparable transfection efficiency, expansion and phenotype, but with reduced cytotoxicity, compared to traditional viral plasmids. **C** T cell engineering can be performed and assisted by NPs in vivo instead of standard ex vivo manufacturing. For example, CAR or TCR encoding mRNA, as well as TCR activating/expanding factors can be delivered to T cells in vivo using lipid nanoparticles. Site-specific T cell targeting can be achieved by surface anchoring NPs with specific antibodies

Cancerous cells develop diverse mechanisms to bypass immune surveillance and establish immune tolerance, together enabling tumor progression [90]. Numerous immunotherapeutics targeting cancer immune evasion have emerged in the last half-century; the two most prolific being immune checkpoint (IC) inhibition therapies and adoptive cellular therapies (ACT). Lipid-based nanosystems have been explored for improvements to such therapies (Fig. 3). Considering the advantages of NPs mentioned above, the primary rationales for using NPs in immunotherapies are enhanced, combined and potentially controlled delivery of immunomodulatory reagents.

### Immune checkpoint inhibition nanosystems

Immune checkpoints represent a group of surface molecules that maintain physiological homeostasis in immune self-tolerance through modulation of T cells, natural killer (NK) cells, and MDSCs upon activation, and are exploited in tumor immune evasion [91]. Inhibiting these checkpoints reactivates the immune system to mount a robust, highly specific response against cancer cells, leading to wide therapeutic success [92, 93]. Monoclonal antibodies (mAbs) are the most popular agents for IC therapy, also termed immune checkpoint inhibitors (ICIs), but many new IC therapeutic agents, including NP-based, small-molecule inhibitors and gene therapies have been or are being developed (Table 1) [94–98].

**Table 1:** Clinically used immune checkpoint inhibitor antibodies and preclinical lipid-based NP examples targeting immune checkpoints

IC target	Clinical antibodies		Preclinical applications in combination with nanosystems				
	Name	Ref	Nanoparticle type	Conjugates and/or payloads	Target	Effects	Ref
CTLA-4	Ipilimumab, Tremelimumab	[94, 96, 99–101]	Liposomes	Doxorubicin and anti-CTLA-4 antibodies encapsulated in hydrophilic core	Effector T cells	anti-CTLA-4 administered before Doxil showed synergism in both non-liposomal and liposomal forms and increased the CD8^+^/Treg ratio	[102]
			Liposomes	anti-CTLA-4 antibodies encapsulated in hydrophilic core	Effector T cells	Longer half-life, higher survival and tumor inhibition	[103]
PD-1	Nivolumab, Pembrolizumab, Cemiplimab, Dostarlimab, Spartalizumab,	[93, 95, 104–118]	ZnGa2 O4:Cr3 + core, TiO2 shell loaded in a Liposome	anti-PD-1, paclitaxel	Tumor cells and effector T cells	Penetration through blood–brain-barrier for accumulation in glioblastoma, ROS-mediated drug release, tumor killing, inflammation, enhanced survival, and long-term immuno-surveillance	[119]
			Liposomes	ROS-sensitive paclitaxel derivative, small molecule PD-1/PD-L1 inhibitor (BMS-202)	Tumor cells and effector T cells	Superior antitumor activity, paclitaxel-mediated ICD, sustained ICI response, recovered host immune surveillance, high co-loading ability	[120]
			Cationic thermosensitive LNPs	Photosensitizer IR-780 and small molecule PD-1/PD-L1 inhibitor (BMS-202)	TME and tumor cells	Deeper tumor penetration by NPs after reducing cancer-associated fibroblasts and increasing the number of tumor-infiltrating lymphocytes	[121]
			Liposomes modified with mannose and hyaluronic acid	CpG ODNs, PD-L1 antagonistic peptides	Tumor cells, macrophages and effector T cells	Improved antitumor immunity, reversal of M2/M1 macrophage polarization, targeted anti-tumor activity, systemic circulation stability	[122]
			Liposome microneedles	anti-PD-1, cisplatin	Effector T cells	Increases effector T cell activity, cytotoxicity of cisplatin and inhibits tumor growth	[123]
			Thermosensitive magnetoliposomes	Doxorubicin, PD-1, imaging agent (iron oxide NPs)	Tumor cells and effector T cells	Doxorubicin burst-release on demand, superior magnetic resonance imaging, potentiated by PD-1 blockade	[124]
			Cellular membrane vesicles	PD-1 molecule, IDO inhibitor	Tumor cells and T cells	Increased infiltrating T cells	[125]
PD-L1	Atezolizumab, Avelumab, Durvalumab	[126–134]	Liposomes	anti-PD-L1 (Fab'), doxorubicin	Tumor cells	Dual activity: Increases Dox efficacy and activation of effector T cells (20% tumor regression in mice)	[56, 57]
			Liposomes	anti-PD-L1, CSF1R inhibitor	Macrophages	TAMs repolarization to M1-like phenotype, increases the numbers of tumor-infiltrating T cells and induces tumor growth control	[95]
			Liposomes	PD-L1 binding peptides, doxorubicin	Colon cancer	PD-L1 is trafficked towards the degradative lysosome, immune escape reversed, doxorubicin is released, tumor reduced	[135]
			Lipid gel depot	Photothermal agent IR820 and anti-PD-L1	Tumor cells and effector T cells	Sensitizing immunologically cold tumors to ICI through PTT, recruitment of tumor-infiltrating lymphocytes, enhanced T cell activity against tumors	[136]
			Liposomal nanohybrid cerasomes	anti-PD-L1, paclitaxel, IRDye800C, gadolinium	Tumor cells	Dual imaging capabilities and antitumor efficacy (theranostic)	[96]
			Liposomes	anti-PD-L1 conjugated, docetaxel loaded	Tumor cells	Low systemic toxicity, induced tumor accumulation, in vitro and in vivo antitumor effect, tumor-specific CD8^+^ T cell activation, and prolonged survival	[137]
			Liposomes	PTT agent (IR780), folic acid-linked oxaliplatin (OXA) prodrug, PD-L1 inhibitors (BMS-1)	Tumor cells	Enhanced PTT effect, prolonged circulation, increased tumor accumulation, induction of ICD, PD-1/PD-L1 blockade, and immune activation against rechallenge and metastasis	[137]
			Enzyme and pH-sensitive micelle-liposome double-layered particles	Paclitaxel, thioridazine, and anti-PD-1/anti-PD-L1 HY19991	Tumor cells	Enhanced T cell infiltration, high drug concentrations in tumors, significant (> 93%) anticancer efficacy,	[138]
			Liposomes	Surface anti-PD-L1, loaded with irinotecan, JQ1 (small molecule oncogene inhibitor)	Tumor cells	Induction of ICD, increased CD8 + /Treg ratio, increased interferon-gamma (IFN-γ), tumor reduction	[139]
			Liposomes	anti-PD-L1, dinaciclib	TAMs	TAM depletion	[140]
			LNPs	mRNA encoding XA-1 (bispecific PD-1 and PD-L1 antibodies)	Tumor cells and effector T cells	Increased AUC over freely injected XA-1, enhancing antitumor efficacy	[141]
LAG-3	IMP321 (fusion protein), Relatlimab (BMS-986016)	[142, 143]	–	–	–	–	–
TIGIT	Vibostolimab (MK-7684), AB154, Tiragolumab, BMS-986207, Etigilimab (MPH313), ASP8374, Ociperlimab (BGB-A1217)	[144]	Liposomes	Liposome-reconstituted, Fyn-phosphorylated TIGIT intracellular domain or PD-1 intracellular domain	–	PD-1 inhibits CD226 phosphorylation via Shp2 recruitment, while TIGIT did not inhibit pCD226. CD226 deficiency alters CD8^+^ T cell transcription and function	[145]
TIM-3	LY3321367, TSR-022, Sabatolimab (MBG453)	[118, 143]	Blood clot scaffold loaded with liposomal protamine-hyaluronic acid NPs	TIM-3 and PD-L1 siRNA, antigens, adjuvants	T cells and DCs	Formation of an antigen-specific DC-rich environment, reduction of immunosuppressive signals from mature DCs, T cell priming, also for personalized therapy with neoantigens	[146]
B7-H3	Enoblituzumab	[147]	LNPs	Anti-B7H3-CD3 bispecific antibody mRNA as bispecific T cell engagers (BiTE)	Tumor cells	Targeting ability towards hepatosplenic region, prolonged half-life in vivo, lasting antitumor effects	[148]
IDO1	Indoximod, Navoximod (NLG919), BMS-986205, Epacadostat		Liposomes (redox responsive)	Porphyrin-phospholipid conjugate, IDO inhibitor	Tumor cells	Induction of ICD, reversal of suppressive TME, tumor accumulation and prolonged circulation, PDT activity, inhibition of tumor growth with reduced phototoxicity, lymphocyte infiltration	[149, 150]
			Liposomes	Protoporphyrin IX (PpIX), NLG919	Tumor cells and effector T cells	Inhibition of metastasis, PDT and IC-mediated tumor killing, ICD leading to ROS generation and an enhanced immune response, CD8^+^ T cell infiltration, primary and distant tumor inhibition	[151]
			Liposomes	NLG919 and oxaliplatin prodrugs	Tumor cells	Synergistic antitumor efficacy in both subcutaneous and orthotopic tumors, enhanced CD8 + T cell infiltration, prevention of immunosuppression through inhibition of tyrosine-kynurenine conversion	[152]
			Liposomes	Mitoxantrone and a cholesteryl indoximod prodrug	Tumor cells	ICD and cytotoxic cell death, reduced FOXP3^+^ Treg cells, successful co-delivery of agents	[153]
OX40	Rocatinlimab	[154]	–	–	–	–	–
OX40L	Amlitelimab	[154, 155]	LNPs	OX40L-mRNA or OX40L-mRNA in combination with IL-23 and IL-36γ mRNA	Effector T cells	Improved T cell function, expansion and survival. Tumor reduction in immune-infiltrated tumors. Addition of cytokines reduced TME suppression: ~ 50% tumor reduction in 1 dose	[156]
			LNPs	OX40L mRNAs	Tumor cells and APCs	CD4^+^ and CD8 + T cells were significantly increased and survival prolonged	[157]
VISTA	–	[158–160]	Liposome	Biotin-Streptavidin-conjugated VISTA	Lymphocytes	Increased circulation half-life to 60 h, alleviated graft rejection during treatment	[158]
BTLA	–	[161, 162]	–	–	–	–	–

*IC* immune checkpoint, *CTLA-4* cytotoxic T lymphocyte-associated antigen, *PD-1* programmed death-1, *PD-L1* programmed death ligand-1, *LAG-3* lymphocyte activation gene 3, *TIGIT* T cell Ig and ITIM domain, *TIM-3*T cell Ig and mucin domain 3, *B7-H3* CD276, *IDO1* indoleamine 2,3-dioxygenase-1, OX40, CD134 or Tumor necrosis factor super family 4 (TNFRSF4), OX40L, OX40 ligand, *VISTA* V-domain Ig-containing suppressor of T cell activation or B7-H5 (PD-1H), *BTLA* B- and T-lymphocyte attenuator or CD272, *AUC* area under the curve, *PDT* Photodynamic therapy, *CpG ODN* cytosine-phosphorothioate-guanine oligodeoxynucleotides, *DC* dendritic cell, *APC* Antigen-presenting cell, *PTT* Photothermal therapy, *ROS* reactive oxygen species, *ICD* immunogenic cell death, *TAM* tumor-associated macrophage, *TME* tumor microenvironment, *NP* nanoparticle, *IL* interleukin

Although ICI-mAbs have drastically changed the landscape of cancer treatment, clinical response rates, especially in solid tumors, are relatively low [33, 94, 163]. This is partially due to low penetration and premature removal from circulation [37]. Further, between 10 and 40% of patients (monotherapy) are likely to experience severe (grade 3/4) immune-related adverse events (irAEs) [96, 164, 165], 0.3–1.2% of which could be fatal (grade 5) [166–168], forcing discontinuation of therapy. These challenges in ICI dosing have led the focus in ICI research to shift towards the mechanism of response and resistance-based studies, as well as the identification of rational combinations to increase safety and efficacy, with over 5000 clinical trials approved at the time of writing. These trials evaluate combinations of ICIs with drugs, NPs, and other ICIs, with 83% combined with chemotherapy, radiotherapy, or other immune therapies [95]. The areas in which ICI treatment can be assisted by the unique properties of lipid-based NPs are further investigated in this section (Table 1).

#### Clinical and preclinical research

Significant benefits have been reported in the literature for IC-blocking and IC-targeted NPs, in both in vitro and murine tumor models, over freely administered IC therapeutics [37]. In step with IC therapy in the clinic, NPs targeted toward cytotoxic T lymphocyte-associated antigen-4 (CTLA-4), programmed death-1, and its ligand (PD-1 and PD-L1) have been the most prominent (Table 1) [37]. For enhanced accumulation in tumor regions and reduced toxicities, one popular strategy is hiding ICIs in the liposomal hydrophilic core. For instance, anti-CTLA-4 mAbs encapsulated inside the PEGylated liposomes, showed superior capacity for tumor reduction, and favorably changed the CD8^+^ effector T cell to Treg ratios in mice bearing C26 colon tumors, when compared to free anti-CTLA-4 [103]. Li et al. [119] loaded anti-PD-1 mAbs into the core of liposomes and paclitaxel into the hydrophobic lipid bilayer, with luminescent nanosensitizers and sonosensitizers in the same NP for imaging monitoring with externally controlled characteristics. These combinations led to deeper penetration through the blood‐brain‐barrier, local release of anti-PD1 mAbs and paclitaxel, thus enhancing the survival rate of glioblastoma-bearing mice [119]. Moreover, ICIs have also been widely decorated on the surface of NPs for both immune checkpoint-blocking and specific cell-targeting purposes. For instance, He et al. [139] developed a liposome targeting anti-PD-L1 via surface conjugation, with irinotecan and JQ1 (a small molecule inhibitor of oncogenes) in the inner core. In this system, the mAbs can target PD-L1 overexpressing cancer cells, thus, accumulation of irinotecan and JQ1 at the tumor site can be increased [139]. As shown by the examples above, the remarkable advantage is the synergistic activity of different therapeutic approaches which can be achieved via a single NP. A useful example of this is the synergistic combination of ICD-inducing chemotherapeutic agents, e.g., doxorubicin, daunorubicin, and paclitaxel, with immunotherapeutics (details shown in Table 1) [120, 137, 139, 149–151, 169].

As an extension to the concept of mAbs in NPs, other types of antibody fragments are also being actively investigated. Surface conjugation of the anti-PD-L1 antibody Fab’ fragments on PEGylated liposome surfaces was demonstrated to show more rapid tumor accumulation, superior circulation time, and an increased number of tumor-specific CD8+ T cells compared to liposomes bearing whole mAb molecules (with a higher area under the curve in 24 h in serum). This Fab’-conjugated LPs resulted in total tumor regression in 20% of mice, which was also not observed in the whole-mAb group [56]. In subsequent studies, the encapsulation stability of co-loaded doxorubicin was enhanced by the liposomal surface decoration of anti-PD-L1 antibody Fab’ [57]. Other researchers have also utilized the PD-1/PD-L1 axis by developing liposomes decorated with multivalent PD-L1-binding peptides, which promoted lysosomal degradation of the PD-L1 molecule on tumor cells upon binding, alleviating PD-L1 mediated T cell suppression [135], or using proprietary small molecule PD1/PD-L1 inhibitors, for instance, BMS-202 [120].

IC therapies have also been achieved via non-antibody-related methods, such as siRNA-mediated knockdown [146] or checkpoint ligand-encoding mRNA delivery mediated by NPs [141, 170]. These are innovative methods for achieving similar outcomes and have been delved into in later sections of this paper. Other ICs, e.g., lymphocyte activation gene 3 (LAG-3), T cell Ig and mucin domain 3 (TIM-3), and T cell Ig and ITIM domain (TIGIT) have been targeted by NPs with promising results being reported for many combinations of free inhibitors of these newer ICs, especially with PD-1 or PD-L1 (Table 1) [171–174].

Nanotechnology offers attractive advantages for tackling the known limitations of IC therapies. These advantages include reduced systemic dosage, enhanced tumor accumulation, selective or triggered cargo delivery, and the flexibility to incorporate different types of molecules in the core or on the surface of NPs. Particularly in combination therapies, dual activity through co-presentation in time and space maximizes the potential for synergy, improving therapeutic outcomes [32, 34, 48].

### Adoptive cellular therapy nanosystems

ACT generally refers to treatment with cancer-specific T cells. These T cells are isolated from a patient, in some cases genetically modified to recognize tumor-specific targets, expanded ex vivo, and then transfused back into the same patient (Fig. 3B) [175]. For hematological malignancies, the use of T cells gene-engineered with antigen-specific T cell receptors (TCRs) and chimeric antigen receptors (CARs) has been highly effective: six CAR-T cell therapies have been approved by the FDA, four being anti-CD19 CARs, and the other two targeting B-cell maturation antigen [176]. The clinical success of such therapies in solid tumors; however, has been low to moderate at best, due in part to the low levels of unique cancer antigen expression in solid tumors, introducing risks for destructive targeting of healthy tissues [33, 177, 178]. In comparison to IC therapy, where many NP combination therapies have been considered [179], potential NP-ACT combinations remain relatively unexplored. Here, we describe how NPs have been utilized in ACT engineering (Fig. 3B) and combined with ACT in preclinical models and early clinical trials to potentiate anti-tumor T cell responses (Fig. 3C). Three approaches are discussed, including rationales, examples, expectations, and challenges, where NPs enable: (1) gene-engineering of T cells; (2) activation of T cells via cytokine delivery, and (3) surface-tethering of T cells.

#### Nanoparticles for in vitro and in vivo T cell gene engineering

NPs can improve the efficiency of gene transfer to T cells. For instance in vitro, they have been reported to successfully deliver engineered TCR-encoding mRNA, as well as mRNA encoding many other genes in a virus-free system, and to lower toxicity and improve T cell function [180]. Billingsley et al. [181] screened a library of 24 ionizable lipids for LNP production to improve mRNA delivery. The most proficient transfection lipid (denoted C14-4) showed reduced cytotoxicity, and comparable transfection efficiency (compared to electroporation) for mRNA produced from CD19-CAR lentiviral vector plasmid, and yielded CAR T cells that were able to kill Nalm-6 lymphoblastic leukemia cells [181]. Similarly, Ye et al. [182] developed an efficient CAR T cell in vitro transcription platform based on LNPs, which also successfully created T cells for the selective killing of B cell lymphoma cells. These novel approaches underscore the importance and capabilities of rationally designed LNPs for nucleic acid delivery going forward. What’s more, Lu et al. [183], who applied a liposome-encapsulated Clustered Regularly Interspaced Short Palindromic Repeats (CRISPR) and CRISPR-associated protein-9 nuclease (Cas9) (CRISPR/Cas9) genome editing method to knock out the PD-1 gene from T cells. These PD-1^−^ T cells reduced tumor growth in HepG2 xenografts [183].

The in vivo generation of anti-tumor T cells using NPs would potentially be a welcome step in the field of ACT as it may circumvent the ex vivo manufacture of T cells, which has proven a significant burden with respect to the time and costs of preparing a clinical product. Zhou et al. [184] have demonstrated the successful in vivo generation of leukemia-specific CAR T cells using CD3^+^ T cell-targeted, nucleic acid-loaded LNPs. These LNPs comprised (1) surface-anchored anti-CD3 antibodies to enable T cell targeting, and (2) a plasmid containing a disease-specific CD19-CAR and an IL-6 short hairpin RNA (shRNA) for knockdown of IL-6 expression, to prevent cytokine release syndrome. This novel approach resulted in successful in vivo engineering of anti-CD19 CAR T cells, with around 84.1% CAR^+^ CD3^+^ T cells on day 21 after treatment, compared to a lower CAR expression ratio, 56.4%, in the conventional IL-6^+^ ex vivo CAR T cell control group. This treatment resulted in a similar overall survival time and less toxicity in a murine leukemia model compared to traditional CAR-T cells [184]. In another example, LNPs were targeted by Rurik et al. [185] to CD5^+^ lymphocytes, enabling expression of a CAR specific for fibroblast activation protein, thereby reducing fibrosis and improving cardiac function in a murine heart failure model. Lastly, LNPs have recently been employed for the first time to enable gene editing in the clinic [186]. Patients with transthyretin amyloidosis were treated by delivering complexed with Cas9-encoding mRNA and a single guide RNA targeting transthyretin, resulting in an 87% reduction in serum transthyretin levels.

Collectively, these examples are paving the way towards increased usage of NPs for in vitro or in vivo T cell engineering. Such an application does not need to be restricted to T cells. In fact, it has already been expanded to adoptive macrophage and NK cell therapy, where CAR macrophages and CAR/TCR-NK cells have been generated [182, 187, 188]. NP-engineered ACTs have achieved high specificity and the potential to overcome immunosuppressive signaling, both alone and in combination with other NP-based and immune therapies, necessitating the use of a suitable delivery platform.

#### Nanoparticle-mediated interleukin delivery for enhancement of T cell function

Cytokines are protein molecules with central roles in T cell functioning [189]. Interleukin (IL)-2, IL-12 and IL-15 are three T cell-stimulating cytokines explored for their usage in ACT through systemic administration of recombinant proteins. However, dose-limiting severe adverse effects and short circulation half-lives generally limit their use without a delivery system [190].

NP-mediated delivery of IL-2 at the tumor site has been employed to enhance T cell activity [191]. In one study, a hybrid lipid system was used for co-delivery of IL-2 and an inhibitor of transforming growth factor-beta (TGF-β). Features from liposomal and polymer systems cooperated, leading to simultaneous release of both hydrophobic inhibitors and hydrophilic cytokines [191]. Delivery of both agents reduced B16 melanoma tumor growth and enriched T cells and NK cells in mice, compared to NPs loaded with only IL-2 [191]. IL-2 itself has also been utilized to target liposomes to T cells [192]. Here, an engineered IL-2 was decorated onto the surface of a PEGylated liposome, which showed higher binding to ACT-T cells compared with endogenous T cells in the lymph nodes, blood and spleen. This IL-2 receptor targeting liposomes, and not free IL-2, allowed specific and repeated targeting, and expansion of ACT-T cells in B16F10 tumor-bearing mice [192].

IL-12 has also been delivered locally using NP-based approaches [172, 173, 193]. Intra-tumoral administration of IL-12-encoding mRNA, using diamino lipid-containing LNPs, suppressed tumor growth in a melanoma mouse model, where further suppression was observed upon the addition of IL-27 mRNA into the same LNPs for co-administration [194]. Delivery of LNPs carrying viral self-replicating RNA coding for IL-12 in B16F10 melanoma, combined with anti-PD-1 therapy, also resulted in significant delays to tumor growth and extended survival when compared to anti-PD-1 therapy alone [195]. Importantly, in the same model, retention of IL-12 was observed when RNA for this cytokine was linked to RNA coding the collagen-binding protein lumican. Buffering of IL-12 through collagen-binding further eliminated lung metastasis formation upon systemically injected tumor cell rechallenge [195]. These studies demonstrate that the local NP-mediated induction of IL-12 can induce a systemic response.

In extension to these findings, tumor growth control has also been observed in murine hepatocellular carcinoma and melanoma models upon intravenous (i.v.) administration of LNPs containing mRNA coding for IL-12 fused to a collagen-binding domain [196]. Furthermore, these studies reported IL-12-induced enhancement of CD8^+^ T cell infiltration and intra-tumoral IFN-γ production [194–197]. Notably, IL-12 has the ability to induce differentiation of type 1 helper (CD4^+^) T cells, and to activate NK cells, NK T cells, and CD8^+^ T cells, which may contribute to the abscopal effects observed with NP-IL-12 treatments. For example, local injection of IL-12 mRNA-loaded LNPs into the tumor resulted in regression of treated as well as distal non-treated tumors, while concentrations of circulating IL-12 did not increase due to the production of IL-12 in the treated tumor [172]. Further, a combination of IL-12 mRNA-LNPs with anti-PD-L1 mAbs significantly and synergistically enhanced the shrinkage of tumors, survival, T cell infiltration, and IFN-γ production [172]. Preliminary clinical results in patients with diverse cancer types treated with IL-12 mRNA-LNPs (MEDI1191) in combination with durvalumab (anti-PD-1; NCT03946800) indicate safety, lack of irAEs, and antitumor activity, with approximately 30% of treated candidates showing stable disease or partial response [198]. Given these safety indications, a follow-up clinical approach can be a dose escalation study of intratumoral MEDI1191 in patients receiving ACT to enhance the ACT efficacy.

#### Surface tethering of T cells for delivery

Adoptively transferred T cells themselves can also serve as vehicles to deliver NPs specifically to the tumor site, which would not only deliver agents at the site where T cells are going, but also enhance the function of T cells at the site of delivery. The redox status of the TME can affect T cells’ function through altering the balance between the –SH and S–S groups on their surface. Therefore, using reagents that neutralize reactive oxygen species (ROS) could help to combat this effect on T cells. Shi et al. [199] pretreated T cells with anti-CD3-coupled fusogenic liposomes, which served as “competitors” of T cell oxidation, resulting in T cell activation and tumor regression in a panel of murine cancer models. Similarly, decorating the surface of CD19 CAR-T cells ex vivo prior to systemic administration with multilamellar liposome-encapsulated adenosine inhibitor SCH-58261 via maleimide functionalization and surface thiol binding, enhanced their function in a mouse ovarian cancer model [200]. Moreover, functionalized liposomes can be loaded on ACT T cells directly in blood by targeting their specific surface ligands. As such, ACT T cells as well as liposomal drugs could reach tumor sites simultaneously without clearance by the MPS. For instance, systemic injection of anti-Thy1 liposomal TGF-β inhibitor after pmel-1 Thy1.1^+^ CD8^+^ T cell therapy in a B16F10 tumor model showed better tumor regression and total survival rates compared to ACT T cells with and without free drug [201].

Other cytokine therapies including, IL-15 super agonist (IL-15Sa)/IL-21, together with the glycogen synthase kinase 3-beta inhibitor TWS119, have also been encapsulated into multilamellar lipid-based NPs yielding a stable conjugation to T cells. Interestingly, this platform has also been applied to other reagents and adjuvants, and potentially enables the delivery of a diverse range of agents along with T cells or other “vehicle” cells [202]. Nanogels encapsulating IL-15Sa that are coupled to T cells via an anti-CD45 antibody have already been tested in combination with concavalin A-primed CAR T cells [203]. This combination treatment resulted in a higher local dosage of IL-15, induction of CAR T cell expansion, and improved in vivo tumor clearance when compared to systemic administration of free IL-15 and CAR T cells [203]. Similar findings have been reported for IL-2 tethered to adoptively transferred T cells [204]. The latter study made use of redox-responsive IL2/Fc nanogels and relied on an increase in T cell surface redox activity upon stimulation, enabling antigen-dependent release of IL-2. Adoptively transferred gp-100 TCR T cells conjugated to these NPs expanded more, demonstrating higher activity and better tumor control in a murine B16F10 melanoma metastasis model, with even lower toxicity, when compared to ACT combined with systemically administered IL-2 [204]. Other biocompatible nanosystems, including biocompatible hydrogels and microparticles, have also been co-delivered with T cells to improve their retention and activation, resulting in increased T cell expansion and tumor reduction compared to T cells administered without [205–208].

Overall, NPs can be used for engineering T cells, activation of T cells via delivery of cytokines, or enable the use of T cells as vehicles (through conjugation); and for all these technical applications, studies have shown clear preclinical and clinical evidence of potentiation of anti-tumor T cell responses. In combination with ACT, these NP-based technologies have shown benefits including both enhanced efficacy and reduced toxicity. In the view of the authors, it is only a matter of time before such combinational therapies are tested in clinical settings [203, 204].

### Lipid-based nanoparticles in clinical trials

Lipid-based NP-immunotherapies have shown promise in translational in vivo studies, and subsequently, their clinical use is expanding. Table 2 presents a summary of representative liposomes, LNPs, and polymeric NPs in clinical oncology trials at the time of writing (excluding the vast number of chemical agent-NP compositions).

**Table 2:** Liposomal and lipid nanoparticle formulations for cancer therapy currently in clinical trials

Drug	Cancer type	Biological payloads	NCT number	Phase
Liposomes				
SGT-53	Solid tumors Breast cancer	Wild type p53 sequence plasmid, Anti-transferrin receptor single-chain antibody fragment	NCT02354547, NCT05093387	1 1
DOTAP:Chol-FUS1 Liposomes	Solid tumors NSCLC	TUSC2 plasmid	NCT00059605, NCT01455389	1 1/2
EGFR antisense DNA and DC-Chol liposomes*	Squamous head and neck cancer	EGFR antisense DNA	NCT00009841	1
MRX34	Solid or hematologic tumors	miRNA-34a	NCT01829971 NCT02862145	1 1/2
LErafAON	Solid tumors	Antisense Oligonucleotide c-raf	NCT00024661 NCT00024648	1 1
BP1001-A**	Solid tumors	Antisense Oligonucleotide Grb2	NCT04196257 NCT02923986	1 1/2
BP1001	Chronic myelogenous Leukemia acute myeloid leukemia Myelodysplastic syndrome	Antisense Oligonucleotide Grb2	NCT02923986 NCT02781883 NCT01159028	1/2 2 1
PNT2258	Solid tumors	DNA oligonucleotide (target the regulatory region upstream of the BCL2 gene)	NCT01191775	1
BP1002	Acute myeloid leukemia	Antisense Oligodeoxynucleotide BCL-2	NCT05190471	1
EphA2-targeting DOPC-encapsulated siRNA	Solid tumors	EphA2 siRNA	NCT01591356	1
NA-LPs	Pediatric gliomas Glioblastoma	Autologous total tumor mRNA, pp65 full-length LAMP mRNA	NCT04573140	1
DPX-0907	Ovarian cancer Breast cancer Prostate cancer	Seven tumor-specific HLA-A2-restricted epitopes tumor-associated antigens, Topoisomerase II alpha, B-cell receptor-associated protein 31 (CDM protein), TACE, Abl2, Gamma catenin (Junction plakoglobin), EDDR1, Integrin beta 8 subunit	NCT01095848	1
W_ova1 vaccine	Ovarian cancer	3 OC TAA RNAs	NCT04163094	1
Tumor-specific RNA-NP vaccine	Melanoma	Autologous total tumor mRNA	NCT05264974	1
L-BLP25	Prostate cancer NSCLS Multiple myeloma Breast cancer	Synthetic lipopeptide derived from the mucin 1, Adjuvant MPLA	NCT01496131 NCT00157209 NCT01094548 NCT00157196 NCT01423760 NCT00925548 NCT00409188	2 2 2 2 NA 3 3
ONT-10	Solid tumors	Synthetic glycolipopeptide MUC1 antigen, M40Tn6, Synthetic TLR-4 agonist, PET Lipid A	NCT01556789	1
Autologous tumor cell vaccine	Follicular lymphomas	Autologous tumor-derived antigen	NCT00020462	1
PDS0101	Cervical cancer	R-DOTAP (Versamune) to boost the immune system's response against the HPV viral proteins, Selected peptides HPV	NCT04580771 NCT05232851	2 1/2
Lipovaxin-MM	Melanoma	Tumor antigens (gp100, tyrosinase, and MART-1), DC-targeting moiety, DMS-5000, which is a DC-SIGN-specific, VH domain antibody fragment DC-maturing cytokine, interferon gamma (IFN-γ)	NCT01052142	1
Anti-EGFR immunoliposomes loaded with doxorubicin	Solid tumors	Anti-EGFR antibody Doxorubicin	NCT01702129	1
MBP-426	Gastric cancer Esophageal cancer	Oxaliplatin Anti-transferrin receptor antibody	NCT00964080	1/2
C-VISA BikDD	Pancreatic cancer	BikDD, a phosphorylation mimic mutant form of pro-apoptotic protein Bik	NCT00968604	1
Liposomal interleukin 2	Melanoma	IL-2	NCT00004104	2
*Lipid nanoparticles*				
INT-1B3	Solid tumors	miRNA-193a-3p	NCT04675996	1
WGI-0301	Solid tumors	Antisense Oligonucleotide Akt-1	NCT05267899	1
mRNA-2416	Solid tumors or lymphoma	mRNA encoding for the OX40L protein	NCT03323398	1/2
mRNA-2752	Solid tumors or lymphoma	mRNA encoding for OX40L, IL-23, and IL-36γ	NCT03739931	1
MT-302	Solid tumors	TROP2-FcA mRNA	NCT05969041	1
MEDI1191	Solid tumors	IL-12 mRNA	NCT03946800	1
OTX-2002	Hepatocellular carcinoma and other solid tumor types known for association with the MYC oncogene	mRNA encoding for ZF-DNMT and ZF-KRAB proteins	NCT05497453	1/2
DCR-MYC	Solid or hematological tumors	MYC siRNA	NCT02110563, NCT02314052	1 1/2
ALN-VSP02	Solid tumors	KSP and VEGF siRNAs	NCT01158079	1
TKM 080301	Solid tumors	PLK1 siRNA	NCT01437007, NCT01262235	1 1/2
Quaratusugene Ozeplasmid	SCLS, NSCLC	TUSC2 plasmid	NCT05703971, NCT05062980	1/2 1/2
*Hybrid (lipid and polymer) nanoparticles*				
EGEN-001 Lipopolymer	Ovarian epithelial cancer Fallopian tube cancer Primary peritoneal cancer	IL-12 plasmid	NCT01489371 NCT01118052	1 2
*Polymeric nanoparticles*				
NKTR-255	Large B-cell lymphoma	IL-15 receptor agonist	NCT05664217	2/3
CA102N	Solid Tumors Colorectal Cancer	Hyaluronic acid, nimesulide	NCT03616574 NCT06039202	1 2
CRL 1005	Melanoma	No payload, as independent adjuvant	NCT00003274	2

*IL* interleukin, *NSCLC* non-small cell lung cancer, *TUSC2* tumor suppressor candidate 2, *DOTAP* 1,2-dioleoyl-3-trimethylammonium-propane, *EGFR* epidermal growth factor receptor, *miRNA* micro ribonucleic acid, *Chol* cholesterol, *c-raf* RAF proto-oncogene serine/threonine-protein kinase, *Grb2* growth factor receptor bound protein 2, *BCL2* B-cell lymphoma 2, *EphA2* ephrin type-A receptor 2, *DOPC* 1,2-dioleoyl-sn-glycero-3-phosphocholine, *siRNA* small interfering ribonucleic acid, *LP* lipid nanoparticle, *mRNA* messenger ribonucleic acid, *LAMP* lysosomal associated membrane protein, *TAAs* tumor-associated antigens, *TACE* TNF-alpha-converting enzyme, *Abl2* abelson homolog 2, *EDDR1* epithelial discoidin domain receptor 1, *NP* nanoparticle, *MPLA* monophosphoryl lipid A, *TLR-4* toll-like receptor 4, *HPV* human papillomavirus, *MART-1* melanoma antigen recognized by T cells, *AKT-1* AKT serine/threonine kinase 1, *OX40L* TNF ligand superfamily member 4, *TROP2* trophoblast cell-surface antigen 2, *MYC* master regulator of cell metabolism, *DNMT* DNA methyltransferase, *KRAB* Krüppel-associated box, *KSP* kinesin spindle protein, *PLK1* polo like kinase 1, *SCLS* small cell lung cancer. *BP1001-A, Bio-Path’s lead drug candidate, prexigebersen modified to produce smaller drug nanoparticles; **DC-cholesterol, is a derivative of cholesterol

Clinical trial applications are mainly focused on cancer vaccines and targeting tumor progression, either by enhancing the expression of tumor-suppressive genes or silencing oncogenes. NPs can shield payloads (antigen/adjuvant) from the interacting biofluids, enhance their half-life and biocompatibility, facilitate their accumulation in antigen-presenting cells (APCs), and induce the priming of T cells. There are several lipid-based NP-vaccines being tested in clinical trials, e.g., DPX-0907 (NCT01095848), W_ova1 vaccine (NCT04163094), L-BLP25 (NCT01496131 and others; Table 2), and ONT-10 (NCT01556789). Furthermore, personalized immunotherapy can be achieved by NP-mediated encapsulation of autologous antigens (NCT04573140, NCT05264974, NCT00020462). Gene delivery to tumors is another extensively explored strategy, leveraging the significant advancements in the field of gene therapy. LNPs harness distinct advantages over other systems with their high loading capacity, intrinsic pH-sensitive properties facilitating endosomal escape, and ease of scalability. A variety of gene types, including DNA, siRNA, mRNA, and antisense oligonucleotides, have been encapsulated within LNPs and evaluated in clinical trials (Table 2). Additionally, numerous clinical trials explore combination strategies within a single NP formulation, as opposed to monotherapies. For instance, SGT-53 utilizes a liposome comprising p53 plasmids along with an anti-transferrin receptor single-chain antibody fragment (NCT02354547, NCT05093387). Similarly, ALN-VSP02 incorporates KSP and VEGF siRNAs within the same LNP (NCT01158079).

Direct targeting of the immune system is another lipid-based therapeutic that prominently emerged in the clinic in recent years, with a number of NP-mediated IL delivery therapies being tested in the clinic. EGEN-001, consisting of a human IL-12 plasmid and PEG-polyethyleneimine-cholesterol lipopolymer was evaluated in phase 1 and 2 trials in patients with recurrent ovarian cancer. These studies demonstrated feasibility and safety, and revealed some, albeit limited, activity of EGEN-001 (no complete or partial response, stable disease in 35%) and appeared to indicate higher toxicity in platinum-resistant patients (NCT01118052) [209]. In another phase 1 trial of EGEN-001 in combination with liposomal doxorubicin, increased levels of IL-12, IFN-γ, and TNF-α were observed in peritoneal fluid following administration (NCT01489371) [210]. The highest proportions of partial responders (28.6%) and stable disease (57.1%) were found at dose level 3 (the maximum tolerated dose was not reached). Another tested therapeutic is mRNA-2752, a lipid NP encapsulating mRNAs encoding human OX40L, IL-23, and IL-36γ [156, 172]. This novel therapeutic was administrated intratumorally as monotherapy or in combination with durvalumab in patients with advanced solid malignancies or lymphoma. Results demonstrated that mRNA-2752 could induce the expression of IL-23, IL-36γ, IL-22, IL-6, IFN-γ, TNF-α, and PD-L1, and cause tumor shrinkage [211]. Currently, enrollment is ongoing in expansion cohorts focusing on triple-negative breast cancer, urothelial cancer, lymphoma, ICI-refractory melanoma, and non-small cell lung cancer (NCT03739931).

Another widely explored non-lipid-based NP system is polymeric NPs. Similar to lipid-based NPs, the majority of clinical evaluations have focused on incorporating chemotherapy agents within these NPs. There have also been trials related to active targeting (e.g., NCT03616574) and cytokine delivery (e.g., NCT05664217). Although there are currently not as many clinical trials as lipid-based NPs, we expect the advancements in materials science and chemistry to accelerate the developments in this field.

In summary, lipid-based immunotherapy primarily aims at gene delivery of cytokines, often in conjunction with other therapies such as chemotherapy or monoclonal antibody therapy. With the growing understanding of immunotherapy, emergence of novel materials, and the increasing success of combination therapies in clinical settings, more advanced multi-functional NP applications building upon above mentioned innovative approaches are expected to emerge in the future. This evolution signifies the progression towards the next generation of anti-tumor immunotherapies, where NPs play a pivotal role in enhancing treatment efficacy and precision.

## Targeting the suppressive tumor microenvironment with lipid-based nanosystems

**Fig. 4:**
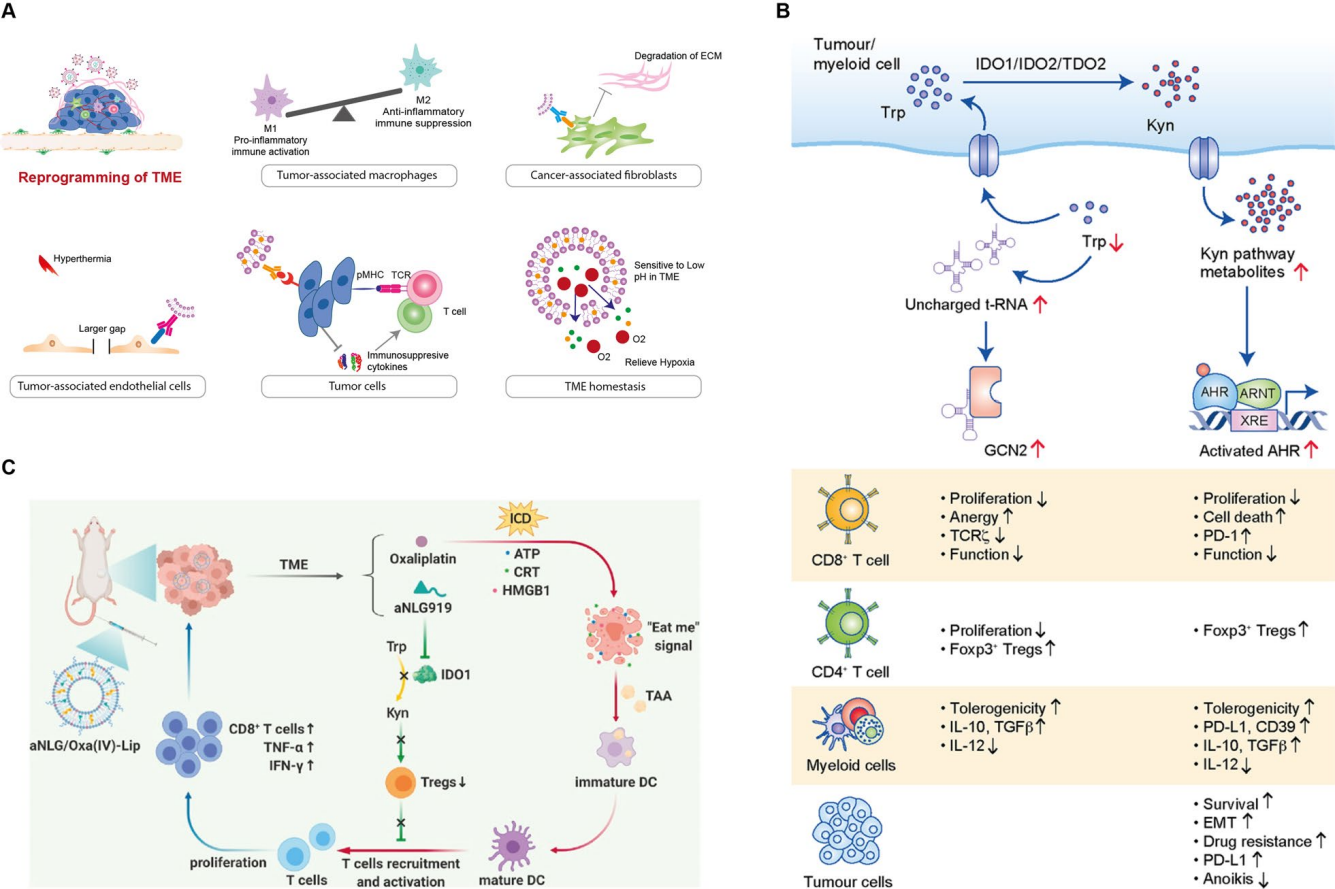
Targeting the suppressive tumor microenvironment with lipid-based nanosystems. **A** Strategies that utilize lipid-based nanoparticles to enhance anti-tumor immune responses through modulation of the tumor microenvironment (TME) include: (1) Targeting innate immunosuppressive immune cells, such as tumor-associated macrophages, to induce their polarization from M2 to M1 phenotype. (2) Targeting stromal cells, such as cancer-associated fibroblasts for degrading the extracellular matrix to facilitate deep and homogeneous NP penetration into the TME. (3) Overcoming the vasculature barrier by actively targeting endothelial cells or inducing extrinsic factors like hyperthermia to improve the enhanced permeability and retention effect. (4) Sensitizing tumor cells to immune cell attack by reducing immunosuppressive cytokines and inducing tumor-specific antigen presentation, and (5) Modulating the hypoxic TME by delivering molecules such as hemoglobin to alleviate hypoxia-associated immunosuppression and to improve anti-tumor immune responses. **B** The immunosuppressive role mode of action and effects of (1) tryptophan (Trp) depletion and (2) kynurenine (Kyn) increase, on cells in the TME. Specific effects on CD8^+^ and CD4^+^ T cells, myeloid cells and tumor cells are shown. **C** Schematic illustrating the synergistic therapeutic efficacy of bifunctional aNLG/Oxa(IV)-Lip [152]. Upon intravenous injection, aNLG/Oxa(IV)-Lip will release cytotoxic oxaliplatin inside the reductive cytosol to induce cancer death via the ICD pathway and thus release the damage-associated molecular patterns to promote DC maturation and prime the host’s immune system. In the meanwhile, it would release NLG919 to inhibit the IDO1-mediated conversion of essential Trp to immunosuppressive Kyn decreasing the frequency of immunosuppressive Tregs inside the tumors and promoting the intratumoral infiltration of CD8^+^ T cells and secretion of anti-tumor cytokines TNF-α and IFN-γ. This results in the inhibition of residual tumor growth. **B** Reproduced with permission on the basis of a CC-BY open access license [213]. **C** Reprinted from Shen F et al. with permission from Elsevier [152]

In solid tumors, the immunosuppressive TME remains one of the key challenges limiting the success of immunotherapy. In this section, we present the crucial benefits that lipid-based NPs can offer to improve the interactions between immune cells, non-immune stromal cells, and tumor cells in the TME. Strategies for TME-targeting encompass both the reversal of inhibitory signals and the enhancement of stimulatory signals [212].The most extensively investigated approaches further discussed here include: (1) Reversal of inhibitory signals and enhancement of stimulatory signals in immune-associated innate immune cells; (2) Sensitization of tumor cells by reducing immunosuppressive cytokines, inducing tumor-specific antigen presentation, blocking immune checkpoints (discussed in “Delivery capabilities” Section), or generating immunosuppressive metabolites; (3) Targeting stromal cells and remodeling the physical barriers in drug delivery; and (4) Combining the above approaches with modalities like photodynamic therapy (PDT) and chemotherapy for synergistic effects (Fig. 4A).

### Interaction between tumor microenvironment and lipid-based NPs

Here, we focus on discussing the targeting of three representative cell types within the TME: (1) tumor-associated macrophages (TAMs), (2) tumor cells, and (3) cancer-associated fibroblasts (CAFs) [214, 215].

Macrophages are recruited into the TME from the circulation [216, 217] and most become polarized towards the pro-tumor M2 phenotype, enhancing tumor cell proliferation, angiogenesis, neovascularization, metastasis, and immune suppression [218, 219]. Strategies to reduce TAM presence in the TME or preferably to re-polarize M2-type TAMs to the antitumor M1 phenotype, have been developed to improve the treatment of solid tumors [220]. One of the first macrophage-targeting liposome formulations was the large multilamellar clodronate liposomes, developed by van Rooijen in the 1990s [221], which efficiently depleted macrophages in different organs and tissues [222]. More recently, Fritz et al. [223] used clodronate-loaded liposomes to selectively kill macrophages in a murine urethane-induced lung adenocarcinoma model. This led to a decrease in total alveolar macrophage populations by more than 50%, and also a 50% reduction of tumor burden [223]. It is worth noting one drawback of clodronate liposomes: these are not macrophage subset-specific. These NPs target M1 and M2 macrophages alike, resulting in a relatively unaffected M1/M2 ratio in the TME [223].

Targeting of extracellular or intracellular signaling pathways in TAMs using NPs is another strategy used to improve the anti-tumor activity of TAMs [224–227]. For example, using siRNA-loaded LNPs, Shobaki et al. [228] attempted to silence the signal transducer and activator of transcription 3 (STAT3) and hypoxia inducible factor 1α (HIF-1α) genes in TAMs. Multiple i.v. administrations in OS-RC-2 renal cell carcinoma xenograft-bearing mice successfully induced anti-tumor immune functionality by increasing the number of intratumoral (CD11b^+^) macrophages and M1 (CD169^+^) macrophages, repolarizing M1/M2 macrophages, and inhibiting tumor growth, compared with the untreated control group. This treatment reversed the tumor-activating and angiogenic functions of TAMs and showed no toxic effects in treated mice [228]. Wang et al. [229] used LNPs loaded with mRNA encoding a bispecific antibody against the chemokines CCL2 and CCL5, responsible for TAM recruitment, infiltration, and M2-polarization [230]. The i.v.-injected LNPs accumulated in the liver and were taken up by Hepa1-6 murine HCC cells (inoculated to the liver via hemispleen injection) and myeloid cells, resulting in effective expression and secretion of the bispecific antibody [229]. This approach achieved successful blockage of CCL2 and CCL5, resulting in decreased macrophage infiltration and repolarization of the existing M2 macrophages toward the M1 phenotype. Survival was also significantly prolonged by more than double that of the control group; even more so when combined with a PD-1 ligand inhibitor. The absence of toxicity was confirmed through function tests of the liver and kidneys, as well as body weight stability [229].

Lipid-based NPs can also be utilized to reduce the secretion of certain immunosuppressive cytokines from tumor cells, such as vascular endothelial growth factor (VEGF), TGF-β and IL-10 [231]. For example, Xu et al. [232] delivered TGF-β siRNA to tumor cells with a liposome-protamine hyaluronic acid NP, where tumor targeting was achieved by coupling anisamide onto the liposomal surface to recognize the sigma receptor overexpressed on tumors. This approach resulted in a 50% knockdown of TGF-β in the tumor tissues of subcutaneous B16F10 murine melanoma-bearing mice, increased CD8^+^ effector T cell infiltration and decreased Treg numbers, compared with using a lipid-based NP vaccine system delivering Trp2 peptides (as tumor antigens) and CpG oligonucleotides (as adjuvants) [232]. NPs can also be used to induce tumor antigen expression, thereby increasing tumor recognition by the immune system. One approach to do so would be via the delivery of chemotherapeutic reagents with NPs to induce ICD, eliminating tumor cells and inducing local adaptive anti-tumor immune responses [10]. The encapsulation capacities of lipid-based NPs for chemotherapeutic agents are well studied, including for those inducing ICD [233–235]. Several drugs are already on the market, and thousands of clinical trials are underway investigating other combinations [36, 236].

Another approach to enhance tumor cell susceptibility to the immune system involves targeting indoleamine 2,3-dioxygenase-1 (IDO1) to address the immune-suppressive microenvironment [237]. IDO1, also mentioned previously as a target for immune checkpoint blockade, operates as an immune suppressor (Fig. 4B) [213]. It is often overexpressed in tumors, degrading tryptophan to kynurenines, which increase immune regulatory and evasion functions, and promote metastasis [237, 238]. This also affects populations of T cells within the TME, with kynurenines increasing T cell anergy and Treg induction, as well as decreasing inflammation and immune infiltration [238, 239]. Suitably, IDO1 overexpression is associated with poor survival in a range of cancers [239–242], but conversely, also with improved survival in certain cases [243], thus, leaving its exact effects and mechanisms thereof in need of elucidation. Several attempts have been made to utilize IDO1 as a drug target in clinical settings [237, 244]. Clinically, IDO1 inhibitors [245], especially indoximod and navoximod (NLG919) have been thoroughly investigated alone and in combination with other therapies such as chemo-, immuno-, and radiotherapy (NCT02835729, NCT03301636, and NCT0546949, respectively, amongst others) [246]. Co-delivery is a key determinant of success when combining these agents with other modalities. Sun et al. [237] designed such a delivery system to co-deliver NLG919 and doxorubicin using polymeric micelles. In this example, a considerable increase in circulation time of the IDO1 inhibitor-micelle over doxorubicin was observed, as well as significantly prolonged survival and tumor reduction over both doxorubicin and liposomal doxorubicin [237]. Indoximod is another therapeutic which has been administered using lipid-based nanocarriers, as carried out by Mei et al. [153], whereby an indoximod prodrug was delivered alongside the chemotherapeutic mitoxantrone in colon and breast cancer models. Following administration, they detected ICD markers calreticulin and high mobility group box 1 protein, alongside perforin and granzyme B, showing cytotoxic and immunogenic cell death with boosted pharmacokinetics and stability thanks to cholesterol-prodrug conjugation [153]. Other liposomal formulations have been published by Liu et al. [149, 150], Huang et al. [151], and Shen et al. [152] (Fig. 4C). These have been synergistically combined with PDT and ROS-inducing therapies [149–151], as well as chemotherapeutic prodrugs [152], with promising antitumor effects, establishing the groundwork for the future of IDO1-targeted nanotherapies.

Lastly, CAFs promote tumor progression by remodeling the ECM and producing cytokines and growth factors, e.g., IL-6 and stromal cell-derived factor-1 (SDF-1) [247–251], making them potential NP targets to improve immunotherapy efficacy [252]. For example, stromal remodeling was achieved by administering CAF-targeting peptide-modified liposomes delivering captopril, resulting in a reduction of ECM deposition by blocking the TGF-β1-Smad2 related signaling pathway [252]. This effect was utilized in tandem with liposomal gemcitabine treatment, administered subsequently, achieving improved pancreatic tissue penetration [252].

While these multifunctional delivery platforms are excellent tools to simultaneously administer immunomodulatory agents and remodel the TME, targeting of macrophages, fibroblasts, or other TME components should be carefully considered and executed as these interventions may also impact normal functioning. Temporal and spatial control of such therapeutics are needed to circumvent off-target toxicity, poor penetration, and interference with normal tissue homeostasis. NP-mediated drug delivery, as discussed in detail above, can address several of these issues making TME targeting safer and more efficacious.

### Features in the tumor microenvironment hindering nanoparticle delivery

Although TME remodeling by NPs has shown very promising results, there are still several barriers to solid tumor therapy due to the pathophysiology of the TME, which severely limits the delivery of NPs to the tumor. Firstly, the chaotic network of tumor vasculature, overproduction of ECM, proliferation of myofibroblast-like cells, and desmoplasia, all contribute to an increase in tumor interstitial fluid pressure, thereby causing hypo-perfusion of therapeutic agents [253, 254]. Meanwhile, endothelial cells serve as the initial barrier preventing nanoparticles from reaching the tumor site. Passive accumulation of nanoparticles in the TME facilitated by the EPR effect is highly heterogeneous across various tumor types and even within individual tumors [255]. For instance, pancreatic cancer is often characterized by non-leaky blood vessels and dense stroma, resulting in a greatly lowered EPR effect [256]. Here, combinations with vasoactive agents, such as TNF-α, or mechanical triggers, such as hyperthermia, can homogenize and elevate the extravasation of liposomes to increase delivered drug levels toward therapeutic concentrations [257, 258]. The EPR effect is also affected by anti-angiogenic agents such as bevacizumab. Such agents developed to specifically halt growing vessels found in the tumor could actually cause the already existing tumor-associated vasculature to assume a more normal structure. This vessel normalization compromises the extravasation of larger (> 100 nm) NPs, decreasing the EPR effect. At the same time, more normalized vasculature improves blood flow, which is beneficial for radiotherapy and chemotherapy when using free agents and also for smaller NPs like micelles and quantum dots (< 100 nm) [259]. Such effects should be considered on a case-by-case basis.

Given the effects of the intricate interplay between TME elements on tumor immune evasion and NP delivery, combinational immunotherapeutic approaches present opportunities as well as challenges. In general, spatiotemporal dosing control and complex PKs inherent to each agent have presented a formidable obstacle in achieving synergistic effects. Here, the diverse delivery capacities of lipid-based NPs, enabling drug co-delivery, could offer a potential solution. Treatment modalities targeting different sites in the TME simultaneously or sequentially, or the same site with multiple synergistic agents, can be combined in/on NPs. This remains one of the primary rationales for prioritizing NP utilization.

## Advanced and emerging lipid nanosystems in immune therapy

Thus far, the impact of nanosystems on the field of immunotherapy has been highlighted. In this section, the specifics of utilized nanosystems and capabilities thereof are explored. Firstly, the nucleic acid delivery potential of one of the most advanced nanosystems applied in immunotherapy, LNPs, is discussed from a methodological perspective. Next, exemplary cases are explored, where deep multidisciplinary expertise in both oncobiology and formulation science have yielded efficacious immunotherapeutic lipid-based nanoformulations. The next-generation formulations discussed below include stimuli-sensitive formulations, biomimetically functionalized NPs, and extracellular vesicles (EVs).

### Advanced lipid-based NPs for nucleic acid delivery

Nucleic acid-based therapeutics have shown great potential in immunotherapy. These therapeutics can be categorized based on the function of the nucleic acid delivered: immunostimulatory [260], gene-editing [261], gene-regulating [262–264], or nanovaccine engineering [265, 266]. They modulate cancer immunotherapies through the regulation of immune and tumor cells, reversal of immunosuppression in the TME, or delivery of antigens or immunostimulatory nucleic acids acting as agonists, activators, or antagonists [267]. We have briefly mentioned how NP-mediated delivery can be utilized for overcoming barriers to successful immunotherapy in “Background” Section, including inefficient delivery to target sites, rapid degradation, and low cellular uptake. Three of the advantages mentioned before are particularly instrumental in providing significant advantages for nucleic acid delivery. Firstly, nucleic acid therapeutics are encapsulated within a colloidal system, resulting in higher stability. Secondly, the induction of endogenous translation of functional proteins greatly accelerates their clinical application by bypassing protein preparation and purification steps, as well as pharmaceutical formulation and manufacturing. Lastly, and importantly, pH-responsive NP formulations are essential for achieving endosomal escape, preventing the degradation of cargos in the lysosome and availing delivered molecules to cellular machinery located in the cytoplasm. These, together, have caused lipid-based NPs to be one of the preferred methods for nucleic acid delivery in the clinic.

The two most commonly used lipid types for nucleic acid complexing are cationic and ionizable lipids. Cationic lipids contain a hydrophilic head with a stable positive charge. This enables interaction with negatively charged nucleic acids. Examples include DOTAP or 1,2-di-O-octadecenyl-3-trimethylammonium-propane (DOTMA) [36, 268, 269]. Ionizable lipids have a pKa of between 6 and 6.7 and are protonated at pH values below that, which causes them to become positively charged with pH reduction. Several proprietary examples have also been designed for more specific applications, especially enhanced stability in RNA delivery particles [74]. This not only enables interactions with negatively charged nucleic acids, but also gives these lipids pH-sensitivity, enabling higher biocompatibility upon administration in physiological pH and, more importantly, endosomal escape in lower pH subcellular microenvironments [270].

Liposomes were the first generation of non-viral lipid-based NP nucleic acid vectors which consisted partly of cationic lipids. An example is liposomes designed by Nakamura et al. [271], encapsulating the Stimulator of Interferon Gene (STING) agonist, cyclic dinucleotide-GMP. The STING pathway plays a defensive role against viruses by detecting cytosolic dsDNA, the detection of which induces the production of type I interferons and pro-inflammatory cytokines, activates APCs, and primes CD8^+^ T cells for tumor antigen recognition [271]. This approach induced significant anti-tumor and anti-metastatic effects in a murine B16-F10-lung metastasis model with increased type I IFN levels and NK cell recruitment to the lungs [271]. More representative liposome-based nucleic acid delivery and associated therapeutics for achieving better bioavailability and anti-tumor efficacy are exemplified in Table 3, including: (1) Nucleic acids can be loaded in pH-sensitive liposomes by using cationic lipids; (2) site-specific targeting of nucleic acids can be achieved by surface modification with cell or tissue-specific ligands; (3) extrinsic triggers, e.g., ultrasound, can help to deliver nucleic acids precisely, and (4) by co-delivering diverse types of therapeutic agents, a synergistic effect may be obtained. It is expected that this strategy can put forward immense development for controlling cancer.

**Table 3:** Liposome-mediated delivery approaches for multifaceted immunomodulatory nucleic acids

Strategies	Components	Size (nm)	Nucleic acids	Targeted cells	Ref.
pH-sensitive	DOTMA, DOPE	–	RNA encoding CD4^+^ T cell-recognizable neoantigens CT26 PME1, engineered from five highly expressed CT26-specific mutations with strong predicted major histocompatibility complex class II binding capacity	CD4^+^ T cells	[272]
pH-sensitive	DOTMA or DOTAP, helper lipid DOPE or cholesterol	200–400	RNA encoding influenza virus hemagglutinin	Dendritic cells	[273]
Surface modification	Mannosylated Lip100*	–	MART-1 mRNA	Dendritic cells	[274]
Ultrasound triggered	DSPC, DSPE-PEG(2000)-OMe, Perfluoro propane	150–200	IL-12-encoding plasmid	Tumor cells	[275]
Co-delivery	HSPC, DOTAP, Chol, DSPE-PEG2000-cRGD, DSPE-PEG2000, Anemoside B4	180.7 ± 7.3	PD-L1 siRNA	Tumor cells and vasculature	[276]

*DOTMA* 1,2-di-O-octadecenyl-3-trimethylammonium-propane, *DOPE* 1,2-Dioleoyl-sn-glycero-3-phosphoethanolamine, *DOTAP* 1,2-dioleoyl-3-trimethylammonium-propane, *MART-1* melanoma antigen recognized by T cells, *DSPC* 1,2-distearoyl-sn-glycero-3-phosphocholine, DSPE-PEG(2000) 1,2-distearoyl-sn-glycero-3-phosphoethanolamine-*N*-[amino(polyethylene glycol)-2000], HSPC, L-α-phosphatidylcholine; *IL* Interleukin, *siRNA* small interfering RNA, *cRGD* cyclic arginyl–glycyl–aspartic acid peptide, *PD-L1* programed death ligand 1, Anemoside B4 (AB4), the main saponin isolated from the roots of P. chinensis

Even with the successes of liposomal nucleic acid-based therapies, difficulties with endosomal-lysosomal uptake, compartmentalization, and subsequent degradation have persisted [277]. The lack of ability for endosomal escape has somewhat hindered clinical adoption, as this potentiates a lack of potency and modest increases in safety [278]. Therefore, the gene therapy market has been largely dominated by viral vectors in recent years, such as γ-retroviral or lentiviral vectors, despite their complex and costly manufacturing, and concerns regarding the use of therapeutic viral particles, as well as immunogenicity [279]. However, the very recent successes of lipid-based NPs have paved the way for the expansion of LNP-delivered gene therapy applications [280]. These successes include the approval of Onpattro^®^, and the strikingly successful role of the aforementioned mRNA-LNP COVID-19 vaccines BNT162b2 and mRNA-1273 (Comirnaty^®^ and Spikevax^®^, respectively) against the COVID-19 pandemic. The demonstrated proficiency of LNPs as non-viral vectors and advances in microfluidics-based manufacturing thereof have attracted substantial attention for overcoming historical therapeutic delivery and manufacturing barriers.

**Fig. 5:**
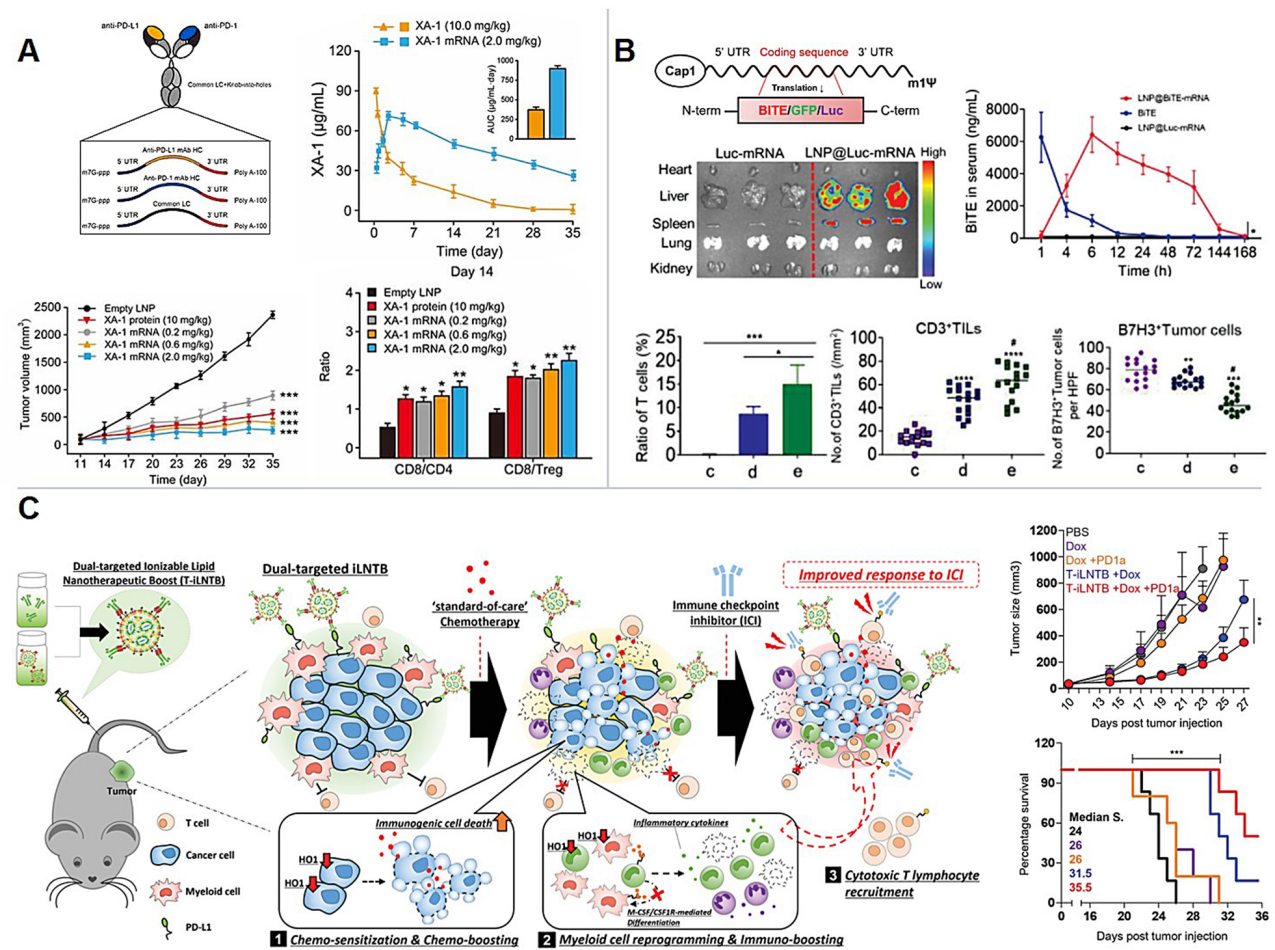
Prominent LNP-mRNA delivery examples from literature. **A** Delivery characteristics of a bivalent antibody (XA-1), or mRNA encoding the same, were compared in terms of circulation time, tumor response, and changes in immune cell composition over time. Prolonged protein production upon mRNA delivery led to a greater antitumor response by increasing the ratios of CD8^+^:CD4^+^ and CD8^+^:Treg T cells [141]. **B** mRNA encoding bispecific T cell engaging (BiTE) antibodies, labeled with luciferase for transcription tracking, was loaded within and delivered via LNPs. This formulation achieved prolonged expression in serum and significantly increased lymphocyte infiltration into tumors (c: 1.5 mg/kg BiTE mRNA + T cells; d: 6 mg/kg BiTE + T cells; e: 1.5 mg/kg LNP@BiTE-mRNA + T cells) [148]. **C** Targeting of LNPs encapsulating siRNA against heme oxygenase-1 (HO1) to tumor and myeloid cells through anti-PD-L1 targeting sensitizes tumors to chemo-immunotherapy, decreasing tumor burden and increasing survival [170]. Abbreviations: *i.v.* intravenous, *PD-1* programmed death-1, *PD-L1* programmed death ligand 1, *PEG* polyethylene glycol, *LNP* lipid nanoparticle, *UTR* untranslated region. ***: *p* = 0.001; **: *p* = 0.01; *: *p* = 0.05. **A**–**C** Reproduced with permission on basis of CC-BY 4.0 [141, 148] and CC-BY-NC [170] open access licenses

These developments have been exploited for nucleic acid delivery in a range of approaches including activation of T cells [281], induction of cancer antigen presentation on cell surfaces (assisting anti-cancer vaccines) [282–286], and signaling pathway modulation [76, 77]. To specifically highlight some of these studies (Fig. 5), Wu et al. [141] engineered LNP-encapsulated mRNA encoding a bispecific anti-PD-1 and PD-L1 antibody (Fig. 5A). The i.v. injection of this formulation showed significantly longer circulation and higher AUC of the antibody when compared to systemic administration of mAbs for the same target. Where the concentration of the freely injected protein in plasma was below 30% of the maximum concentration after seven days, the endogenously translated protein only reached similar levels after 35 days, and the AUC thereof was threefold higher than its freely injected counterpart. Similarly, Huang et al. [148] designed LNPs carrying mRNA encoding bispecific T cell engaging (BiTE) antibodies, tracked in vivo with luciferase labeling (Fig. 5B). They targeted CD3ε on T cells and tumor antigens simultaneously for a potent antitumor effect [148]. Apart from these groups, Yong et al. [170] developed a siRNA-encapsulating LNP against heme oxygenase-1 (HO1), with surface-conjugated anti-PD-L1 mAbs for targeting (Fig. 5C). Treatment of B16F10 melanoma-bearing mice with the targeted siRNA-loaded LNPs in combination with doxorubicin boosted the chemo-immunotherapy efficacy, recruited CD8^+^ T cells, and reduced the number of tumor-promoting M2-like TAMs in the TME. This formulation also assisted in turning the TME to be immunologically “hot”, instead of “cold” (poorly infiltrated by immune cells with low inflammation). Mice treated with a combination of the above LNPs and doxorubicin had approximately 70% lower tumor burdens than those treated with doxorubicin alone [170]. These and other examples given earlier in this paper provide evidence for the capabilities offered by LNPs as a platform, with promising innovation and rapid growth in this field predicted in the foreseeable future.

### Advanced stimuli-responsive formulations

Site-specific drug delivery has attracted interest for the development and regulation of the bioavailability and biosafety of chemotherapeutics and immunotherapeutics alike. Lipid-based NPs have shown the capacity for precise targeting, imaging, and delivery of diverse payloads [287, 288], and can be designed to be responsive to intrinsic (e.g., pH, redox) and extrinsic (e.g., heat, light, and ultrasound) stimuli [75]. This quality, which we have previously defined as “smart,” underscores their sophisticated nature [36].

#### Intrinsic stimuli-sensitive lipid-based nanoparticles

**Fig. 6:**
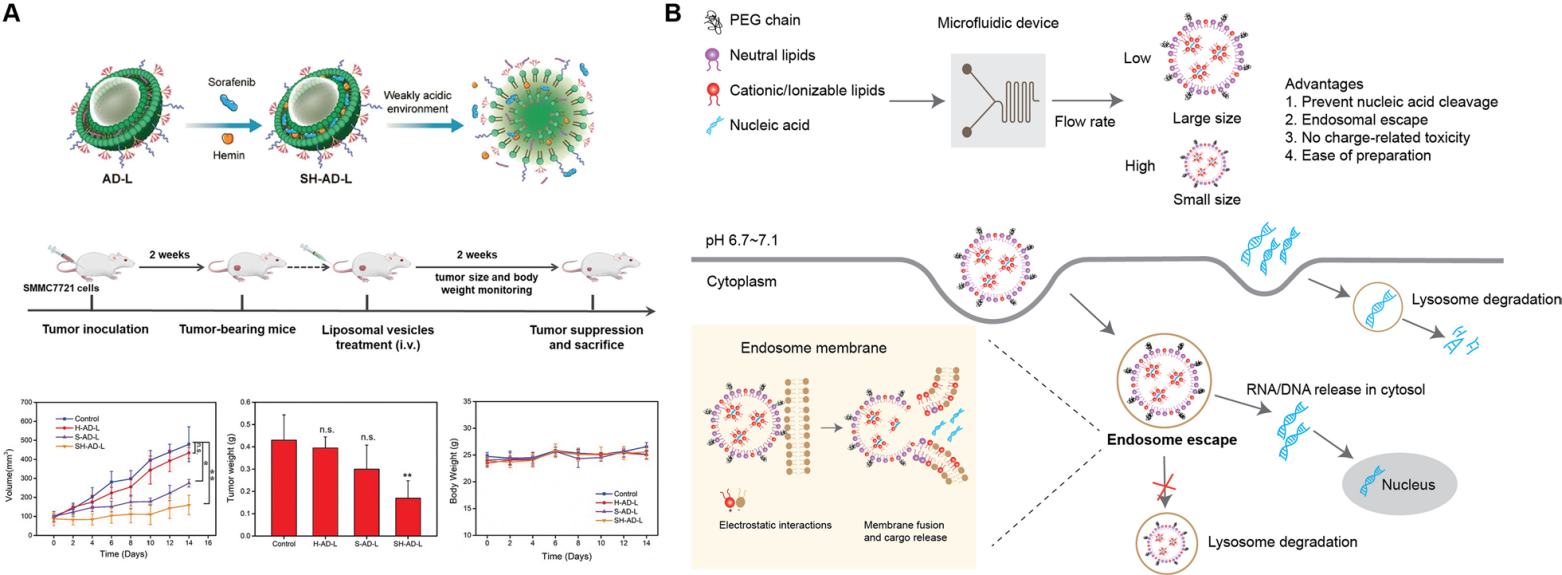
Intrinsic pH-sensitive nanoparticles. **A** Hybrid liposomal vesicles (AD-L) composed of amphiphilic dendrimers (AD) are sensitive to pH change. When entering the weakly acidic TME, sorafenib and hemin encapsulated in the bilayer of AD-L can be released for effective anti-tumor responses in SMMC7721-xenografted nude mice. **B** Compilation of the synthesis and mechanism of action of lipid nanoparticles (LNP)-mediated mRNA delivery. The mechanism by which LNPs achieve successful nucleic acid delivery without degradation of cargo is based on the ionizable characteristics of cationic lipids with a pKa of between 6.0 and 6.7. When cationic, these enable electrostatic interactions with negatively charged nucleic acids at low pH (~ 4.0), ensuring high encapsulation rates. Secondly, these play a crucial role in the escape of intact nucleic acid payloads from the acidic endosomes by membrane fusion into the cytoplasm, where these are translated and become functional. This process categorizes all LNPs, in essence, as stimuli-responsive, triggered-release formulations. Meanwhile, upon administration while exposed to biological fluids (pH 7.4), the surface charge of LNPs remains neutral and unlike permanently cationic lipids, LNPs prepared with ionizable lipids impose no charge-related in vivo toxicity. Ease of preparation, high encapsulation efficiency, and encapsulation yield plus efficient transfection, has made LNPs powerful tools for delivering nucleic acid-based therapeutics for gene therapies. **A** Reproduced with permission, © 2023 Wiley-VCH GmbH [295]

pH-sensitive formulations can be synthesized via different strategies, including combinations of non-bilayer-forming phospholipids and pH-triggered amphiphiles [289, 290], surface modification of lipid-based NPs with pH-triggered groups [291], or incorporation of pH-triggered groups into pH-insensitive biological polymers [75]. In general, the permeability of NP membranes can be adjusted by protonation or deprotonation of pH-sensitive functional groups. The most commonly used example is the natural phospholipid DOPE, which has a bilayer structure at neutral pH but changes to the non-bilayer inverted hexagonal II phase at weakly acidic conditions, resulting in membrane destabilization and cargo release (Fig. 6B) [292, 293]. The benefits of pH-sensitive NPs are mainly based on two aspects. Firstly, the TME is generally slightly more acidic (pH 6.7–7.1) than the normal tissues (pH 7.3–7.4), due to hypoxia and the aerobic glycolysis pathway preference of tumors [294]. Therefore, NP systems responsive to slightly acidic conditions can achieve specific and localized drug release at the TME. Along this line, Su et al. [295] developed a pH-sensitive hybrid liposomal vesicle consisting of liposomes and amphiphilic dendrimers, enabling pH-mediated release of encapsulated sorafenib and hemin upon reaching the TME (Fig. 6A).

Additionally, after cellular uptake, NPs undergo a complex endosomal trafficking pathway, sequentially from early endosomes to recycling endosomes, multivesicular bodies, late endosomes, and lysosomes, and finally are either trafficked back to the cell surface or degraded within the lysosome [296]. Therefore, the usage of pH-sensitive NPs allows endosomal escape, and delivery of biological cargos like proteins and nucleic acids to the cytosol and potentially, to the nucleus (Fig. 6B). Here, Yuba et al. [297] designed phosphatidylcholine (PC) liposomes modified with pH-sensitive 3-methylglutarylated-dextran residues (Mglu-Dex) to enable delivery of antigens into the cytosol of DCs by endosomal escape. This approach achieved antigen delivery and generated effective humoral and cellular immunity in mice [297]. Also, as mentioned in the previous section, the delivery of nucleic-acid-based macromolecules within ionizable/cationic LNPs with endosomal escape has categorized all LNPs, in essence, as intrinsic stimuli-responsive formulations.

Next, redox-responsive NPs are built on the principle of high redox potential differences between the oxidizing extracellular space and the reducing intracellular space. Normally, the concentration of ROS-protective glutathione (GSH) in the blood and the ECM is only one 100th to one 1000th of that of cytoplasm [298], while in tumor cells it can reach 7–10 times higher than in normal tissues. Additionally, mitochondrial dysfunction in tumor cells can result in higher levels of ROS [299]. Along this line, electron-transfer reactions have been exploited for tumor-targeted NP delivery of immunotherapeutics, where disulfide bonds, as one of the most redox-sensitive groups, have been widely used. For example, paclitaxel-loaded redox-sensitive liposomes, mediated by incorporation of disulfide-functionalized PC into the bilayer, have been reported as efficacious, but with increased safety over free drug [300]. Further, it has been shown that redox-sensitive lipid-porphyrin liposomes loaded with an IDO inhibitor, can overturn an immunosuppressive TME by inhibiting the activity of IDO in ROS-producing tumor cells, subsequently turning a tumor immunogenically “hot” by decreasing the anti-inflammatory functions of IDO [237]. This formulation also enabled fluorescent imaging as well as PDT, the latter triggering intratumoral infiltration of CTLs through stimulation of doxorubicin- and PTT-induced ICD in tumor cells, together exemplifying the potential of redox-responsive NP-mediated co-delivery of immunomodulatory molecules and chemotherapeutics in synergistic antitumor therapy [237].

#### Extrinsic stimuli-sensitive lipid-based nanoparticles

While above examples have explored intrinsic stilmuli, cargo release and efficacy enhancements can also be achieved by manipulation of the surrounding environment. Localized hyperthermia is one such method already in wide clinical use for solid tumor treatment. General advantages include inhibition of tumor cell survival and DNA repair, modulation of anti-tumor immune responses, and sensitization to radiation and chemotherapy [301]. Additionally, hyperthermia can improve the accumulation of NPs, through modulation of intertumoral fluid dynamics and enhancement of the EPR effect [302–304]. We have previously observed that the gaps of endothelial lining in the TME can increase to 10 µm under hyperthermia, allowing more liposome extravasation into four tumor types (Murine B16 melanoma, BFS-1 sarcoma, Lewis Lung Carcinoma, and BLM human metastatic melanoma) [301]. This phenomenon can persist up to 8 h after hyperthermia and was absent in normal tissues [70]. Thermosensitive liposomes (TSLs) with hyperthermia-mediated phase-changing functional groups have also been used to induce site-specific drug release at mild hyperthermia (~ 42 °C) [305]. TSLs exploit the lipid bilayer solid-to-liquid transition, based on the phase transition temperature (Tm) of the lipid mixture [302–304]. The lipid bilayer membrane can transfer from a solid gel phase to a liquid-crystalline phase at its Tm, which causes increased permeability, allowing encapsulated small drug molecules to be released [305, 306]. The fine adjustment of temperature-dependent local drug release characteristics can be achieved by adjustments to lipid compositions. For example, in our previous study, a formulation consisting of dipalmitoyl phosphatidylcholine (DPPC, Tm = 41 °C), distearoyl phosphatidylcholine (DSPC, Tm = 54 °C), and 1,2-distearoyl-sn-glycero-3-phosphoethanolamine-*N*-[methoxy(polyethylene glycol)-2000] (DSPE-PEG) at a 75:25:5 molar ratio was able to achieve site-specific drug release at 42 °C [307]. Many TSLs have been exploited for immunotherapy; one such TSL targets toll-like receptor (TLR) 7/8, which is associated with poor patient outcomes and tumor progression, due to its role in production of immunosuppressive cytokines, increased cell proliferation, and resistance to apoptosis [308]. Zhang et al. [309] developed TLR7/8 agonist (resiquimod)-loaded TSLs which released 80% of their cargo within 5 min of exposure to 42 °C. They demonstrated that CD8^+^ T cells and M1/M2 macrophage ratios were elevated in neu-deletion murine breast tumors following local injection with R848-TSLs, combined with systemic anti-PD-1 and local hyperthermia, compared with anti-PD-1 or no-treatment controls. This resulted in 8 out of 11 mice experiencing complete tumor regression over 100 days [309]. It was also noteworthy that none of the cured mice grew tumors after tumor re-inoculation, while 100% of control groups suffered from subsequent tumor burden, indicating immune memory by systemic administration of αPD-1 with R848-TSLs and hyperthermia [309]. Additionally, TSLs can be utilized as photothermal therapy (PTT)-inducing agents by incorporation of heater molecules, i.e., molecules that absorb energy from, e.g., lasers or magnetic fields, and produce heat as a consequence. This was shown using liposomes loaded with heater molecule indocyanine green (ICG) and TLR7 agonist, in combination with low-temperature PTT (laser irradiation, 808 nm) [310]. Treated tumors reached temperatures of 45 °C within 6 min, inducing simultaneous ICD, DC maturation, and infiltration of adaptive immune cells into the tumor upon combination with TLR7 agonist, compared with the non-treated group. Treated mice showed significantly lowered tumor burdens, as well as substantial increases in IL-6 and IFN-γ [310]. These approaches are the first steps toward the development of TSL-mediated cancer immunotherapies which can enhance drug delivery to solid tumors, enabling combination therapy, induction of systemic responses, and reduction in resistance and potential adverse events.

Next, PDT/PTT involves using light in combination with either exogenous or endogenous absorbers to induce cytotoxic ROS or local temperature elevation, respectively. Phototherapies including PDT and PTT can stimulate anti-tumor immune responses by a variety of mechanisms [311, 312]. However, low penetration depth of light sources, hypoxia in solid tumors, and systemic phototoxicity limit their efficiency as monotherapy. Incorporating photoactive molecules into lipid-based nanoparticles (NPs) holds promise for addressing these challenges by leveraging light-induced membrane destabilization and permeabilization. For example, the insertion of porphyrin-phospholipids into stealth liposomal doxorubicin was shown to enhance local phototherapeutic efficacy by enabling light-induced drug release and vasculature permeabilization [313]. Alternatively, the oxidation of unsaturated phospholipids leads to increasing permeability of lipid bilayers and cargo release [314]. Along this line, recently, lipid-based light-sensitive NPs have achieved striking synergistic outcomes in the field of immunotherapy by precise control of immunotherapeutic release, induction of ICD, and reversal of hypoxia in the TME [311]. Some combinations with photosensitizers include immune-metabolic adjuvants [149], NK cell activators [315], and immune stimulatory molecules [149]. For example, Huang et al. [316] developed ICG-loaded light-sensitive liposomes delivered by NK cells, by encapsulating hydrophilic ICG within liposomes, which were then taken up by NK cells. This enhanced perforin and granzyme-mediated tumor killing of NK cells, followed by laser irradiation (wavelength 808 nm) inducing ICG-mediated heat production, ablating the remaining tumor cells [316]. Light-sensitive liposomes can also be loaded with chemotherapeutic and photo-responsive molecules to enable synergistic cancer immunotherapy and phototherapy. In one such composition, unsaturated phospholipids in the liposomes showed light-exposure based drug release, together with enhanced ROS, the primary prerequisite for PDT. Furthermore, ROS generation also resulted in the M2-M1 transformation of TAMs [317], making liposomal theranostic agents promising for synergistic, multimodal cancer immuno-phototherapy [318].

Lastly, ultrasound can be used to mount tumor immune responses by breaking tumors up into debris, releasing immunostimulatory molecules or antigens, or by induction of stress-signaling pathways in tumors, triggering immunogenicity, mostly achieved by high-intensity focused ultrasound and lower intensity ultrasound, respectively [319]. Ultrasound can also be combined with nanosystems for gene or antigen delivery into cells (using ultrasound-mediated bubble-like liposome (micro- or nanobubble) destruction), as well as induction of ICD through sonodynamic therapy [319, 320]. For the former, ultrasound aids in cargo release from microbubbles and the associated force mediates drug/DNA delivery into the cytoplasm of the cells. For example, Hayashi et al. [321] reported that sonoporation (1 MHz input frequency, 0.5  W/cm^2^ output intensity, 30 s exposure) in combination with interferon-β (IFN-β) gene-loaded cationic liposomes, reduced growth of metastatic murine colon cancer. Moreover, several studies have used the combination of microbubbles with ultrasound to improve cancer immunotherapy, using these for delivery of ovalbumin [322], B16BL6-extracted antigens [323], and IL-12 plasmid DNA [275], to achieve cancer immunotherapy. While these results are promising, further investigation into formulation and administration of nanocarriers is needed to optimize ultrasound-assisted gene therapy for various cancers.

### Biomimetic lipid-based nanosystems for immunotherapy

**Fig. 7:**
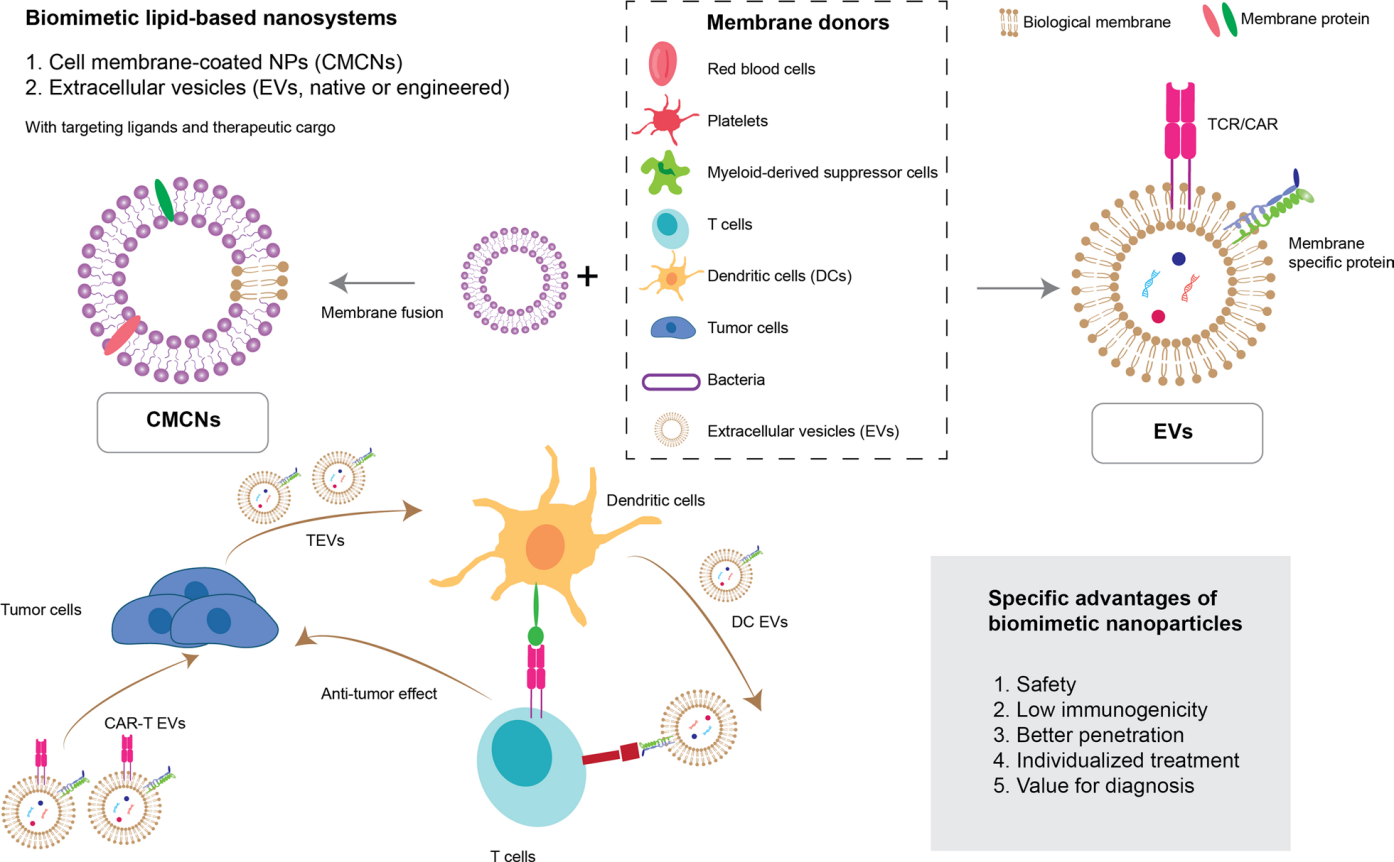
Biomimetic lipid-based nanosystems. These nanosystems include cell membrane-coated nanoparticles (CMCNs) and extracellular vesicles (EVs). CMCNs are synthesized by integrating particular donor cell membranes and traditional lipid-based nanoparticles (NPs). Synthesis approaches include extrusion (membrane fusion), lipid insertion, genetic engineering, and metabolic engineering. Specific EVs have been widely exploited, e.g., tumor cell-derived EVs (TEVs), dendritic cell-derived EVs (DCEVs), and chimeric antigen receptor T cell-derived EVs. These autologous original vesicles contain various endogenous biological information, e.g., specific proteins, membrane receptors, or nucleic acids, which offer great value for delivery of diagnostic and therapeutic payloads. In addition, these systems could also achieve lower toxicities and immunogenicity; longer circulation time and better cell and tissue penetration through increased tropism

Biomimetic NPs, which include cell membrane-coated NPs (CMCNs) and extracellular vesicles (EVs), have recently emerged as both diagnostic and therapeutic tools, with particular strengths for immunotherapy [89], as both retain the macroscopic molecular heterogeneity of the secretor-cells, and in the case of EVs, are natural modes of protein, lipid, and nucleic acid delivery already utilized by cells (Fig. 7) [324]. These endogenously derived, tissue-penetrant, potentially targeted nanosystems have been experimentally applied in diverse anti-cancer immunotherapeutic strategies, especially in strategies utilizing their enhanced tropism towards specific tissues [324–328].

#### Membrane fusion particles

CMCNs may confer biocompatibility, immune-cell co-operation, targeting, and prolonged systemic circulation to lipid-based NPs by integrating cell membrane portions into or onto NPs or surfaces thereof [38, 329]. Cells with unique functional features, e.g., RBCs, platelets, cancer cells, mesenchymal stem cells (MSCs), NK cells, and DCs, have been used as “membrane donors” [38, 330]. Each of these brings unique functionality to a CMCN formulation; the most prominent of which being homotypic targeting (tumor cell membranes), endothelial adhesion and long circulation (immune cell membranes), immune evasion and long circulation (RBC membranes), tumor cell targeting (mesenchymal stem cell membranes), and DC/APC/lymphocyte recruitment and activation (platelets) [89, 331–335]. There has also been much research into developing NPs with access to more specific areas, for example, the blood–brain-barrier [336], and importantly, multiple compartments of the immune system. The effectiveness of CMCNs as alternatives for combination therapy strategies in a safer, tumor-targeted manner are further supported by recent reports that demonstrate improved outcomes of immune therapies [87, 337, 338].

#### EV-based formulations

EVs also put forward many strengths as a nanoplatform, and early on in development, Raposo et al. [339] used lymphocyte-derived EVs carrying MHC and T cell co-stimulatory molecules, to promote antigen-specific MHC II-directed T cell responses in vivo. These vesicles mediated the transfer of peptide/MHC complexes from antigen-exposed APCs to naïve APCs, particularly immature DCs. Along this line, clinical trials testing vaccination with autologous EVs in metastatic melanoma patients demonstrated no significant side effects, primarily supporting large scale production and feasibility of EVs [340, 341]. EVs may also be used in conjunction with other immunotherapeutic modalities, such as CAR-T cell therapies, the primary strengths of which being enhanced intratumoral diffusion and reduced off-target adverse effects [342, 343]. Fu et al. [344] reported higher tumor growth inhibition with CAR-T EVs compared to CAR-T cells with significantly reduced cytokine release syndrome, though potentially lacking some of the T cells’ intracellular signaling and machinery for granzyme production. A similar effect was also shown by Yang et al. [345] with targeted T cell-derived EVs in triple-negative breast cancer, where these EVs, maintaining both TCR and CD3 expression, achieved dose-dependent tumor reduction without side effects. Tumor cell-derived EVs (TEVs), on the other hand, have given rise to a novel way of antitumor vaccination, by interacting with DCs systemically and facilitating endogenous tumor antigen transfer, DC maturation, and increased CD8^+^ T cell recruitment, infiltration, and CD4^+^ T cell memory [346–349].

From these successes, EVs have also raised interest in novel strategies to create semi-artificial particles such as EV-hybrids or EV-biomimetics [350–352]. EV-hybrids consist of EVs modified to incorporate additional synthetic components beyond their natural cargo, commonly through functionalized liposome fusion, whereas EV-biomimetics are fully synthetic EV-like particles mimicking the properties of native EVs [352–354]. Both types retain drug loading and other functionalization possibilities [355]. A key example of a successful outcome in this field was published by Lv et al. [356], whereby CD47-expressing exosomes produced by genetically engineered cells, were fused with TSLs. CD47 enhanced clearance avoidance, enabling the TSL-exosome hybrid to accumulate efficiently in the tumor site following i.v. injection, and release payloads rapidly under hyperthermia. This was combined with docetaxel and granulocyte–macrophage colony-stimulating factor (GM-CSF), leading to macrophage polarization and approximately 25 day increases in survival compared to traditional peritoneal metastases treatment methods [356].

#### Bacterial hybrid formulations

Usage of bacterial cells and membrane materials for immune stimulation has demonstrated these to be attractive emerging tools [357, 358], particularly gut bacteria, which have been shown to increase anti-inflammatory effects, and are able to act as carriers in a range of diseases and routes of administration [359–362]. For instance, gram-negative bacteria-derived outer membrane vesicles (OMVs) have been used as potent immunoadjuvants, with similar functionalization potential [363–365], and interesting unique benefits such as TAM activation and polarization [366], specific CD8^+^ T cell response induction [367, 368], and enhancement of neutrophil-mediated ICD [369].

These findings demonstrate the significant clinical benefits that can be achieved with immunotherapeutic applications of CMCNs, EVs, and hybrid bioformulations, evidently based on conferred biocompatibility and cell targeting capabilities. Ethics and manufacturing of these complex particles are hurdles yet to be addressed, but given the advantages of biomimetic NPs over conventional nanoscale formulations, and this field’s emphasis on rational design and innovation, growth and refinement are expected in the years to come.

## Conclusions, challenges, and future priorities

In this review, we have presented an abundance of evidence supporting the use of lipid-based nanosystems in many forms for diverse applications in cancer immunotherapy, with their unique challenges. Here, we focus on several central caveats that need to be addressed to achieve wider clinical usage of such formulations. Challenges are listed first, followed by potential avenues of improvement upon the current status quo within immunotherapeutic lipid-based NPs.

### Challenges and mitigations: immunotherapeutic lipid-based nanoparticles

*Challenge: Production and manufacturing* remain significant hurdles, especially when combining NPs with complex modalities such as ICI or ACT. The primary issues that remain are scalability and product homogeneity, which both affect the stability and efficacy of NPs, particularly due to difficulties in achieving scalable production protocols for such inherently customizable NPs to pass high-quality control standards.

*Mitigations*:

- Well-characterized isolation, production, and storage protocols are needed, especially to maintain the momentum of such a rapidly developing, experimental field, and its transition into the clinic. Alongside this, the design of new formulations should consider existing and ubiquitous storage and transport methods, which would speed up progression to the clinic.
- Presently, the industry relies on cold-chain transport, posing an obvious problem for countries lacking such infrastructure. Therefore, formulations that do not rely on such infrastructure and can be shipped in a wider range of environmental conditions would help overcome inequitable global distribution and access to life-prolonging immunotherapies. For temperature and humidity-independent transport, lyophilization has been considered, but reconstituted liposomes or LNPs can suffer from poor heterogeneity and inconsistencies, the effects of which remain largely unknown, warranting further research in this area.
- One of the great benefits of using lipid-based NPs is that regulatory hurdles are greatly reduced compared to other classes of nanomedicines. In this case, the closest functional comparison is polymeric nanoparticles. These experience far more regulatory challenges due to widely varying (often novel) formulations being developed and tested [370], whereas there exists widespread and longstanding approval of several lipid-based nanomedicines, shortening their road to market.

*Challenge: Toxicity and off-target effects* of immunotherapeutic lipid-based NPs are relatively well-defined, but should still be considered.

*Mitigations*:

- If unknown, these effects should be characterized for all delivered agents, preferably both individually and as complex formulations, especially in the case of newly modified or developed formulations. A recent example of this is the rare occurrence of acute myocarditis following administration of LNP-based vaccines [371].
- Knowledge of the off-target effects of NP components would streamline the novel uses of approved components, as these have typically already been investigated for safety, efficacy, and sometimes, dosing. The use of immunotherapies and materials used to synthesize NPs that have already received regulatory approval can speed up development. This topic has been mentioned in respective sections of this paper and has also been recently and exhaustively reviewed by Lim et al. [372]. It should be noted that modifications to approved materials, however small, will need to go through the regulatory approval process anew, as these introduce unknown factors, binding sites, and potential new interactions with biological systems, and may cause changes in PD and PK profiles. Studies having led to the approval of the original non-modified materials would act as guidelines.
- Since ICD, while useful for tumor neoantigen presentation and immune cell recruitment, is insufficient as a systemic immune activator, adjuvants or immune-stimulating agents can be used as alternative payloads [31]. These should also undergo comprehensive and individualized toxicity testing to mitigate any risk of increasing unwanted effects, sustaining the advancement of the immunotherapeutic NP field [373].
- Clinically approved commonly used lipids [15, 36] and ionizable lipids [270] will serve as foundations for the development of more LNP-based therapies in the future [374].
- Design of NP combinations with ACT and ICI should consider the common problems of on-target, off-tumor toxicity [375, 376], and off-target toxicity (causing irAEs) [164], respectively. NP-based combination therapies may offer a solution to these issues as their bioaccumulation sites are relatively well-defined [377]. This may reduce unwanted effects from these two types of therapy and provides a strong rationale for NP incorporation.

*Challenge: Cost-effectiveness* is another key point of discussion, and is crucial to the success of immunotherapeutic nanosystems.

*Mitigations*:

- The promise of improved treatment durability of nano-immunotherapies, with fewer total treatments/infusions needed, would decrease hospitalization and financial pressure on the medical system, as well as patient distress, while improving productive, quality life years and eventual societal gain. For treatments such as ACT, in vivo T cell gene manipulation would be a particular breakthrough.
- Lowering side-effects is a key way of reducing pressure on the healthcare system, as patient follow-up is reduced and their lifestyles are improved. Two very successful examples of nanotherapies in the clinic, which have reduced toxicity while retaining the mechanism of action of the delivered drug, are Doxil^®^ and Abraxane^®^ [378].
- The cost–benefit ratio of newly developed formulations will be central to the widespread adoption thereof. Cost-effectiveness and cost–benefit studies have therefore become paramount in informing policymakers and industrial decision-makers on applications, approvals, and reimbursements of existing [379–381] and novel therapies [382] or nanotherapies [383], compared to the current gold standards for particular diseases.
- With the increasing complexity of immunotherapeutic lipid-based NPs, costs of manufacturing and R&D also increase. Thus, the above-mentioned cost-effectiveness gains should be especially prominent if they are to outweigh increased costs.

*Challenge: Customizable and personalized NPs* receiving more attention means that patient characterization/stratification [384], and personalized medicine, might be essential for the sustainable future success of immunotherapeutic lipid-based NPs [36]. Like with all immunotherapies, there is no “one-size-fits-all” therapy.

*Mitigations*:

- For immunotherapy and targeted therapy, this is routinely achieved through ligand or mutation screening prior to treatment [384, 385]. However, with advances in machine learning-based diagnostic models and their wider use in clinical pathology and diagnostics [386], the accuracy of patient response predictions is expected to improve, leading to the increased likelihood of successful treatments, also with NPs.
- At an individual level, increased delivery and clearance evasion can be achieved through the usage of patient material, for instance for the synthesis of biomimetic NPs, or EV fusion-particles.
- For such personalization of therapies, as is for ACT, careful characterization and optimized production are essential to ensuring safety and cost-effectiveness. Particularly for ACT, NPs can be used for faster turnaround of therapy, for instance assisting in the genetic engineering of T cells. For this application, stability is less of a concern than speed, efficiency, and clean results in a Good Manufacturing Practice (GMP) environment. Such applications are a good example of the need to tailor NP formulations to specific needs and goals for the best outcome.

### Priorities for the near future

For the numerous lipid-based nano-immunotherapeutics currently investigated in preclinical and clinical studies, predicting the complex PK/PD profiles of each individual component of multifunctional and combinational treatment strategies remains a priority. The uptake, distribution, content release, delivery, and elimination of nano-immunotherapeutics are all potentially influenced by various factors including particle size, surface charge, targeting ligands, stability, and internal/external payload [36]. This makes it difficult to predict and optimize nanodrug behavior in vivo. Therefore, as with toxicity, it is necessary to define and explore PK profiles of individual and combined formulations [387, 388]. Further, toxicity of newly-designed ionizable lipids needs to be defined more clearly, with a view to adjusting these formulations to mitigate the risk of toxic responses [270, 371, 389].

There has also been much discussion of NPs in vivo being coated with a protein corona from the biological environment, and even traditional surface modification to escape detection and MPS-mediated clearance, like PEGylation, might not be as effective as initially thought [78] This means that the unit eventually interacting with the body is, in practice, the NP with its protein corona. Therefore, the manipulation of protein corona formation to overcome the fluid barrier created by the biological environment has attracted attention, e.g., through incorporation of an artificially prepared corona [390]. Similarly, both EVs and biomimetic lipid-based NPs such as CMCNs are designed for increased compatibility and penetration [89]. Such factors need to be considered and may be areas that could be exploited for enhancement of NP-based therapies in the future.

With the ultimate goal being successful clinical translation, it is crucial to acknowledge the complexity and partially unpredictable behavior of immunotherapeutic NPs, which, together with the limitations of current techniques, may slow the translation of preclinical research outcomes into clinical settings. Therefore, it is essential to maintain a translational and multidisciplinary mindset in the design of new nanosystems, prioritizing clinical applicability by choosing practicality over complexity, and leveraging a range of perspectives and diverse expertise.

## Data Availability

Not applicable; all information in this review can be found in the reference list.

## Abbreviations

ABC: Accelerated blood clearance
ACT: Adoptive cellular therapy
APC: Antigen presenting cell
BiTE: Bispecific T cell engaging
CAF: Cancer-associated fibroblast
CAR: Chimeric antigen receptor
CD: Cluster of differentiation
CMCN: Cell membrane-coated nanoparticle
CTL: Cytotoxic T lymphocyte
CTLA-4: Cytotoxic T lymphocyte antigen-4
cRGD: Cyclic arginyl–glycyl–aspartic acid peptide
CRISPR–Cas9: Clustered regularly interspaced short palindromic repeats (CRISPR) and CRISPR-associated protein-9 nuclease (Cas9)
DNA: Deoxyribonucleic acid
DOPE: 1,2-Dioleoyl-sn-glycero-3-phosphoethanolamine
DOTMA: 1,2-Di-O-octadecenyl-3-trimethylammonium-propane
DOTAP: 1,2-Dioleoyl-3-trimethylammonium-propane
DSPC: 1,2-Distearoyl-sn-glycero-3-phosphocholine
DSPE-PEG(2000): 1,2-Distearoyl-sn-glycero-3-phosphoethanolamine-*N*-[amino(polyethylene glycol)-2000]
ECM: Extracellular matrix
EMEA: European Medicines Agency
EPR: Enhanced permeability and retention
EV: Extracellular vesicle
FDA: Food and Drug Administration
GM-CSF: Granulocyte-macrophage colony-stimulating factor
GMP: Good Manufacturing Practice
GSH: Glutathione
GSK: Glycogen synthase kinase
HCC: Hepatocellular carcinoma
HIF: Hypoxia-inducible factor
HIFU: High-intensity focused ultrasound
HO: Heme oxygenase
HSPC: L-α-phosphatidylcholine
i.v.: Intravenous
LAG-3: Lymphocyte activation gene 3
IC: Immune checkpoint
ICD: Immunogenic cell death
ICEV: Immune cell-derived extracellular vesicle
ICG: Indocyanine green
ICI: Immune checkpoint inhibition
IDO: Indoleamine-2,3-dioxygenase
IFN: Interferon
IL: Interleukin
irAEs: Immune-related adverse events
LNP: Lipid nanoparticle
LPS: Lipopolysaccharide
mAb: Monoclonal antibody
MDSC: Myeloid-derived stem cells
Mglu-Dex: 3-Methylglutarylated-dextran residues
MPS: Mononuclear phagocytic system
mRNA: Messenger ribonucleic acid
MSC: Mesenchymal stem cell
NK: Natural killer
NLC: Nanostructured lipid carrier
NOD2: Nucleotide binding oligomerization domain containing 2
NP: Nanoparticle
OMV: Outer membrane vesicle
PC: Phosphatidylcholine
PD-1: Programed cell death protein 1
PD-L1: Programed death-ligand 1
PDT: Photodynamic therapy
PEG: Polyethylene glycol
PK: Pharmacokinetic
PLGA: Poly lactic-co-glycolic acid
PLT: Platelet
PTT: Photothermal therapy
RBC: Red blood cell
ROS: Reactive oxygen species
SDF: Stromal cell-derived factor
siRNA: Small interfering RNA
shRNA: Short hairpin RNA
SLN: Solid lipid nanoparticle
STAT: Silence the activator of transcription
STING: Stimulator of interferon genes
TAM: Tumor-associated macrophage
TCR: T cell receptor
TEV: Tumor-derived extracellular vesicle
TGF: Transforming growth factor
TIM-3: T cell Ig and mucin domain 3
TIGIT: T cell Ig and ITIM domain
TLR: Toll-like receptor
TME: Tumor microenvironment
TSL: Thermosensitive liposome
TTR: Transthyretin
VEGF: Vascular endothelial growth factor
